# The Multi-Level Mechanism of Action of a Pan-Ras Inhibitor Explains its Antiproliferative Activity on Cetuximab-Resistant Cancer Cells

**DOI:** 10.3389/fmolb.2021.625979

**Published:** 2021-02-17

**Authors:** Renata Tisi, Michela Spinelli, Alessandro Palmioli, Cristina Airoldi, Paolo Cazzaniga, Daniela Besozzi, Marco S. Nobile, Elisa Mazzoleni, Simone Arnhold, Luca De Gioia, Rita Grandori, Francesco Peri, Marco Vanoni, Elena Sacco

**Affiliations:** ^1^ Department of Biotechnology and Biosciences, University of Milan-Bicocca, Milan, Italy; ^2^ SYSBIO-ISBE-IT–Candidate National Node of Italy for ISBE, Research Infrastructure for Systems Biology Europe, Milan, Italy; ^3^ Bicocca Bioinformatics, Biostatistics and Bioimaging Centre - B4, Milano, Italy; ^4^ Department of Industrial Engineering and Innovation Sciences, Eindhoven University of Technology, Eindhoven, Netherlands

**Keywords:** RasG13D, RasG12V, anti-cancer agent, exchange factor, intrinsic nucleotide dissociation and exchange, Raf1 binding, mathematical modeling & simulation, cetuximab

## Abstract

Ras oncoproteins play a crucial role in the onset, maintenance, and progression of the most common and deadly human cancers. Despite extensive research efforts, only a few mutant-specific Ras inhibitors have been reported. We show that cmp4–previously identified as a water-soluble Ras inhibitor– targets multiple steps in the activation and downstream signaling of different Ras mutants and isoforms. Binding of this pan-Ras inhibitor to an extended Switch II pocket on HRas and KRas proteins induces a conformational change that down-regulates intrinsic and GEF-mediated nucleotide dissociation and exchange and effector binding. A mathematical model of the Ras activation cycle predicts that the inhibitor severely reduces the proliferation of different Ras-driven cancer cells, effectively cooperating with Cetuximab to reduce proliferation even of Cetuximab-resistant cancer cell lines. Experimental data confirm the model prediction, indicating that the pan-Ras inhibitor is an appropriate candidate for medicinal chemistry efforts tailored at improving its currently unsatisfactory affinity.

## 1 Introduction

Ras proteins are small guanine nucleotide-binding (G) proteins with low intrinsic GTPase activity, cycling between a GDP-bound inactive state and a GTP-bound active state. They act as molecular switches in signaling pathways regulating many cellular processes, including cell proliferation, growth, survival, adhesion, migration, energy, and redox homeostasis (Simanshu et al., 2017). Ras activity is regulated in response to specific extracellular stimuli, by the competitive action between Guanine nucleotide Exchange Factors (GEFs) promoting the nucleotide dissociation and GDP/GTP exchange, and GTPase Activating Proteins (GAPs), which provide an essential catalytic group for GTP hydrolysis (Scheffzek et al., 1997; Boriack-Sjodin et al., 1998; Bos et al., 2007). In human cells, three *RAS* genes encode four homologous but functionally distinct isoforms (HRas, NRas, and KRas4A and K-Ras4B) (Omerovic et al., 2007; Lu et al., 2016a). Gain-of-function missense mutations, mainly located at codons 12, 13, and 61, constitutively activate Ras proteins and can be detected in approximately one-third of all human cancers. Oncogenic Ras mutants contribute to tumor onset, maintenance, progression, and influence the efficacy of both cytotoxic and targeted therapies (Li et al., 2018). For this reason, many efforts, mostly promoted by the RAS initiative (https://www.cancer.gov/research/key-initiatives/ras), have been devoted to investigating the mechanistic role of *RAS* oncogenes in cancer and to explore different strategies for attenuating the aberrant Ras oncoproteins signaling, as widely reviewed (Sacco et al., 2012c; Welsch et al., 2017; Gorfe and Cho, 2021; Ni et al., 2019; Spencer-Smith and O’Bryan, 2019; Khan et al., 2020; Tisi et al., 2020).

Notably, each oncogenic mutation occurring in *RAS* genes induces conformational changes in the encoded protein that alter the residence time of the protein in the GTP-bound active state (Hunter et al., 2015) and make the oncoprotein surface more or less prone to the functional binding not only with modulators and effectors but also with specific pharmacophore groups or classes of molecule drugs. The Ras^G12V^ mutant presents a weak intrinsic and GAP-mediated GTP hydrolysis, and it is particularly aggressive and refractory to exchange inhibitors (Hunter et al., 2015). We first proved that the Ras^G13D^ mutant shows self-sufficiency in nucleotide dissociation (Palmioli et al., 2009b). Structural and functional studies (Smith et al., 2013; Hunter et al., 2015; Lu et al., 2016b; Johnson et al., 2019; Rabara et al., 2019) indicate that this mutant remains sensitive to the catalytic activity of GEFs and of at least one GAP, Nf1. Active and selective inhibitors for these oncogenic mutants are not yet available. On the contrary, compounds that covalently bind the highly reactive cysteine in the KRas^G12C^ mutant selectively inhibit its function (Ostrem et al., 2013; Lito et al., 2016; Patricelli et al., 2016; Hansen et al., 2018a; Janes et al., 2018). After optimization for clinical use, they show a promising anti-tumor effect in *KRAS* ^*G12C*^-positive patients (Canon et al., 2019; Hallin et al., 2020).

We previously demonstrated that a class of small water-soluble molecules (cmp2-4), specifically binds the Switch II (β-3/α-2) region of wild type HRas-GDP. These compounds inhibit GEF-catalyzed nucleotide exchange, attenuate Ras signaling, and reduce Ras-dependent cell proliferation in mouse fibroblasts (Palmioli et al., 2009a; Sacco et al., 2011). Here we demonstrate that cmp4 binds an extended Switch II pocket on HRas and KRas proteins harboring different mutations. cmp4 decreases the intrinsic and GEF-mediated nucleotide dissociation and exchange on wild type and G13D mutated Ras proteins, interferes with Ras binding to GEFs (RasGRF1 and Sos1) and the Raf1 effector, and reduces mitogen-activated protein kinases signaling and cell viability of KRas^G13D^ cancer cells. A mathematical model of Ras signaling (Stites et al., 2007; McFall et al., 2019), appropriately modified according to recent data (Johnson et al., 2017; Johnson et al., 2019), predicts the ability of cmp4 to inhibit the proliferation of different Ras-driven cancer cells. In keeping with the model prediction, experimental data on human cancer cell lines expressing different Ras oncoproteins confirm that cmp4 is a pan-Ras inhibitor able to cooperate with Cetuximab to inhibit proliferation of Cetuximab-resistant cell lines. Although cmp4 currently has an unsatisfactory affinity for Ras, targeted medicinal chemistry efforts could turn it into a valuable and needed clinical drug.

## 2 Materials and Methods

### 2.1 Compounds and Recombinant

cmp4 was synthesized as described (Palmioli et al., 2009a). Recombinant N-terminal His-tagged wild type and G13D mutated H-Ras proteins (residues 1-166 of the mature protein) and Sos1 catalytic domain (aa553-1024 of the mature protein) were expressed in M15 [pREP4] *E. coli* strain harboring a pQETM-derived plasmid (Qiagen) and purified by affinity chromatography using a Ni^2+^-NTA column (Qiagen), as described (Palmioli et al., 2009b; Palmioli et al., 2017; Sacco et al., 2012a). The N-terminal GST-tagged RasGRF1 catalytic domain (residues 976–1262 of the mature protein), was expressed in BL21 [pLysE] *E.coli* strain harbouring a pGEX2T-derived plasmid and purified by glutathione–sepharose chromatography (Amersham Bioscience) as described (Palmioli et al., 2017).

### 2.2 Mass Spectrometry Experiments

Mass-spectrometry measurements were performed on a hybrid quadrupole-Time-of-Flight (Q-TOF) instrument (QSTAR ELITE, Applied Biosystems, Foster City, CA, United States), equipped with a nano-ESI sample source. Metal-coated borosilicate capillaries (Proxeon, Odense, DK), with medium-length emitter tip of 1-mm internal diameter, were used to infuse the sample. The instrument was calibrated using the renine-inhibitor (1757.9 Da) (Applied Biosystems, Foster City, CA, United States) and its fragment (109.07 Da) as standards. Spectra were acquired in the 1500–3000 m/z range, with accumulation time of 1 s, ion-spray voltage of 1200–1500 V, declustering potential of 80 V, and instrument interface of 50°C. Spectra were averaged over a time period of at least 3 min. Data analysis was performed by the program Analyst QS 2.0 (Applied Biosystems, Foster City, CA, United States). The samples were prepared in 5 mM ammonium acetate pH 6.5.

### 2.3 NMR analysis

For the experiments with the free ligand, cmp4 was dissolved in a [D_11_]-Tris buffer at pH = 7.3, 5 mM MgCl_2_. COSY and HSQC experiments were performed by using the standard sequences. For the binding experiments, wild type or G13D mutated HRas was dissolved in 500 μL of the same [D_11_]-Tris buffer, containing an amount of GDP equimolar to the protein, and transferred into a 5 mm NMR tube; 50 μL of the ligand solution dissolved in the same buffer were added slowly. Final protein concentration was 50 µM, final ligand concentration was 1 mM.

STD experiments were performed without saturation of the residual HDO signal and with spin-lock to avoid the presence of protein resonances in the spectra. A train of Gaussian-shaped pulses of 50 ms each was employed, with a total saturation time of the protein envelope of 2 s. An off-resonance frequency of *δ* = 40 ppm and on-resonance frequency *δ* = −1.5 ppm (protein aliphatic signals region) were applied. Spectra were acquired with a Varian Mercury 400 MHz instrument and processed using the program Mestre-Nova 9.

### 2.4 Flexible Docking Algorithm

Docking analyses were performed in Maestro 10.1 suite (Schrӧdinger) (https://www.schrodinger.com/citations#Maestro). All docking calculations were performed using the Glide software (Glide, version 6.7, Schrödinger, LLC, New York, NY, 2015). The receptor-based molecular docking was carried forward after preparing ligands and proteins as suggested by the developer’s protocols. For HRas and KRas, the pockets corresponding to the residues identified by experimental data on HRas were used as the input for grid receptor definition in induced-fit docking (IFD) workflow with flexible ligand option. The protocol generates alternative cmp4 poses not considering clashes with amino acids side-chains, then optimize the structures obtained by allowing the protein to undergo sidechain or backbone movements during the process (Schrödinger Suite 2015-2 Induced Fit Docking protocol; Glide version 6.7, Schrödinger, LLC, New York, NY, 2015; Prime version 4.0, Schrödinger, LLC, New York, NY, 2015). The IFD extended sampling protocol was employed, generating up to 20 poses per ligand on each iteration. The OPLS 2005 force field (Jorgensen et al., 1996) was used for the minimisation stage, in which residues within 10 Å of each ligand pose were optimised. All other parameters were set to their default values. GLIDE molecular docking output GScore (empirical scoring function) is reported, which is calculated by calculating ligand–protein interaction energies, root mean square deviation (RMSD), hydrogen bonds, hydrophobic interactions, internal energy, π–π stacking interactions, and desolvation. GLIDE Emodel was used to choose the best pose for the ligand in each structure, while IFD Score is based on the Prime calculation of energy content of the structure, and also considers the strain in the receptor and ligands.

### 2.5 Dissociation and Exchange Reactions

Intrinsic and GEF-mediated dissociation and exchange of mant-guanine nucleotides (mant-GXP, GXP being GDP or GTP; Molecular Probes; Invitrogen) assays were performed essentially as described in (Lenzen et al., 1995; Sacco et al., 2012b). Briefly, for dissociation reactions HRas protein was pre-loaded with mant-GXP by incubating for 30 min 250 µM HRas with 750 µM mant-GXP in 40 mM Hepes pH 7.5, 1 mM MgCl_2_, 2.5 mM DTE, 20 mM EDTA. Then 30 mM MgCl_2_ was added and the solution was incubated for further 30 min. Free nucleotides were removed by gel filtration using PD10 desalting columns (Amersham Bioscience) equilibrated with Lenzen buffer (40 mM Hepes, pH 7.5, 5 mM DTE, 10 mM MgCl_2_), and HRas-mant-GXP complex was concentered using centricon 10 KDa (Merck Millipore). The exchange reactions on Ras protein were performed by adding directly in an UV-cuvette 0.25 μM HRas-GXP, and an opportune concentration of cmp4 in Lenzen buffer. After 300 seconds of incubation, a 5-fold excess of mant-GXP (1.25 μM) and a specific concentration of the exchange factor (0 or 0.0625 µM as indicated) were added. The fluorescence measurements were carried out at 25°C using a LS45 fluorescence spectrometer (Perkin-Elmer) with an excitation wavelength of 366 nm and emission wavelength of 442 nm. The reactions were monitored for at least 1500 s. The dissociation reactions were performed in a UV-cuvette by adding to 0.25 μM HRas-mant-GXP, preincubated for 300 s with the opportune concentration of cmp4, 200 μM GXP and a specific concentration of the exchange factor (0, 0.0125, 0.025, 0.0416, 0.125 −0.25 µM), as indicated. Exchange data were fitted to a nonlinear “growth-sigmoidal Hill” curve (*n* = 1), while dissociation data were fitted to an “Exponential decay” curve, using the OriginPro 8.0 software (OriginLab Corporation, MA United States). The initial exchange or dissociation rate for each reaction (initial slope) was determined by computing the first derivative at time zero of the corresponding fitted curves. In the graphs, the maximum value of relative fluorescence (100 on Y-axis) represents the fully loaded Ras status obtained as a start point in dissociation reaction and plateau of an exchange curve obtained in the absence of cmp4.

To measure the affinity for entering nucleotide, a plate-based GDP/GTP titration assay was adapted from the method previously described (Ostrem et al., 2013): 1 µM HRas-mant-GDP complex was added to 96-well black plates in 40 mM Hepes, pH 7.5, 5 mM DTE, 1 mM MgCl_2_. The fluorescence was measured on a Variant Cary Eclipse fluorescence spectrometer (Agilent), with 360 nm excitation and 440 nm emission, before and after 2 h incubation at 25°C with a 5 mM EDTA solution with different concentrations of GDP or GTP, as indicated.

Results for each nucleotide were fitted to a sigmoidal curve using the OriginPro 8.0 software.

### 2.6 Surface Plasmon Resonance Analysis and G-LISA

Surface Plasmon Resonance experiments were carried out by using a BIAcoreX system (BIAcore, GEHealthcare). His-tagged HRas-GDP was immobilized onto a NTA-sensor chip surface (carboxymethylated dextran matrix pre-immobilized with NTA; BIAcore, GEHealthcare), obtaining a surface density of about 4500 resonance units. No nickel solution was injected over the reference cell. The binding with GST-fused RasGRF1 was monitored in real time in the presence of increasing concentrations of cmp4 (0-500 µM). All experiments were performed in HBS-P+ buffer (BIAcore, GE Healthcare) at a flow rate of 10 µL/min. Surface regeneration was accomplished by injecting EDTA (350 mM) in the flowing buffer (30 s contact) two or three times. The evaluation of binding kinetics was performed by using the Biaevaluation software, v. 3.0 (BIAore) and by considering a 1:1 Langmuir interaction. Notably, the value of k_off_ measured in the SPR experiments cannot correspond to the physiological dissociation constants because the absence of free nucleotide in the experiments substantially affects this parameter.

Ras G-LISA Activation assay kit (Cytoskeleton, Inc. BK131) was used to measure the levels of HRas-GTP bound to the Ras binding domain of Raf1 (RBD-Raf1) in the presence of increasing concentrations of cmp4 (range 0.08-500 μM). HRas-GTP 0.4 nM was preincubated in batch with cmp4 for 5 min at RT and then transferred in 96-well coated with RBD-Raf1. After incubation at 4°C for 30 min, the plate was washed three times with washing buffer before the addition of antigen-presenting buffer. The captured HRas-GTP was incubated with the anti-Ras antibody followed by HRP-conjugated secondary antibody. Ras activity was quantified by measuring absorbance at 490 nm.

### 2.7 Cell Lines and Proliferation Assay

Human breast cancer cell line MDA-MB-231, obtained from the American Type Culture Collection, was routinely grown at 37°C in a humidified atmosphere of 5% CO_2_ in Dulbecco’s modified Eagle's medium (D-MEM) (Sigma D6429) supplemented with 10% Newborn Calf Serum (NCS), 2 mM glutamine, 100 units/ml penicillin and 100 mg/ml streptomycin. Human colon adenocarcinoma cell line SW48 (*KRAS* ^*WT/WT*^) and the isogenic SW48 expressing heterozygous KRas^G13D^ (*KRAS* ^*WT/G13D*^) or KRas^G12V^ (*KRAS* ^*WT/G12V*^) were obtained from Horizon Discovery Ltd. Cells were cultured in humidified atmosphere of 5% CO_2_ at 37°C in RPMI 1640 (Sigma R0883) supplemented with 10% Fetal Bovine Serum, 2 mM glutamine, 100 units/ml penicillin and 100 mg/ml streptomycin. Cells were passaged using trypsin–EDTA.

For growth kinetics and RealTime-Glo^TM^ MT Cell Viability Assay (Promega, #G9713) cells were plated into respectively 6-well or 96-well flat-bottomed culture plates at the density of 3000 cells/cm^2^. At 18h after seeding, predetermined concentrations of cmp4 (or water) were added to the cell culture. After 24, 48, and 72 h from treatment, cells were harvested and counted by Coulter Counter to obtain growth curves or treated with 500 X NanoLuciferase and 500 X MT cell viability substrate. The luminescence at different time points after treatment was recorded by using a Victor Multilabel Plate Reader (Perkin Elmer). The viability of cells treated with increasing concentrations of cmp4 was tested relative to the viability of the same cells treated with vehicle (water). Viability results were analyzed by using OriginPro 8.0 software and a nonlinear growth/sigmoidal Hill curve (*n* = 1) to calculate the relative IC50 values.

### 2.8 MAPK Activity

Breast cancer MDA-MB231 were plated (6000 cells/cm^2^) in 60-mm tissue culture dishes. After 18 h different concentrations of cmp4 (or vehicle) were added to the cell culture. After 48 h from treatment, both plate-adherent and in suspension cells were harvested in lysis buffer from PathSscan Sandwich ELISA kit (Cell Signaling). The detection of endogenous levels of Phospho-p44/42 MAPK was performed according to manufacturer's instructions, and the results were normalized on total protein content measured by Bradford analysis.

### 2.9 Mathematical Model

The computational analysis was performed starting from the mechanistic model presented in McFall et al. (2019), where a system of Ordinary Differential Equations (ODEs) is introduced to describe the Ras signaling network. The system of ODEs corresponds to the reactions reported in Supplementary Table S1, where reactions R_1_-R_8_ follow the mass-action kinetics, while reactions R_9_, R_10_, R_11_ follow the Michaelis-Menten kinetics; reaction R_9_ describes the GAP activity, while reactions R_10_ and R_11_ describe the GEF activity. The 11 reactions can be used to simulate both the wild type and mutant proteins by assuming different values of the kinetic parameters. In particular, the kinetic parameters of Ras^G13D^ and Ras^G12V^ mutants were obtained by scaling the wild type parameters (4^th^ column) according to the corresponding alpha factors reported in the 5^th^ and 6^th^ columns of Supplementary Table S2. The scaling factors of Ras^G13D^, related to reaction R_9_, were modified according to the results presented in (Johnson et al., 2019).

Specifically, the computational investigation presented in this work was performed with COPASI (Hoops et al., 2006) (version 4.27), exploiting the LSODA numerical integrator (Petzold, 1983). LSODA is an efficient simulation algorithm capable of dealing with stiff systems by automatically switching between explicit (the Adams’ method) and implicit integration methods (backward differentiation formulae). The accuracy in the description of the solution of the system of ODEs is controlled by the relative tolerance, that is the maximum error allowed in the solution, and absolute tolerance, which is the maximum error allowed in case the solution approaches zero. In the simulations performed here, we considered the following setting: relative tolerance 1e-6, absolute tolerance 1e-12, maximum number of steps executed to generate the solution, at each iteration, 1e5. COPASI was also exploited to perform a parameter sweep analysis (PSA) to investigate the effect of the parameter variations on the emergent dynamics and on the steady-state values of pivotal components of the model. The simulations concerning the PSA have been run by generating a set of different initial conditions for the model, considering a fixed range of variation of the parameter under investigation, and then executing the corresponding simulations with LSODA. In particular, the PSA was performed by varying a single kinetic parameter, considering a logarithmic sampling of values within the specified range. The responsiveness of the Ras^G13D^ mutant variant to GEF activity was analyzed by performing a PSA where the V_max_ of GEF-mediated exchange reactions (R_10_ and R_11_) was multiplied for a parameter gamma, which varied in the range 0-1, where the top value represents the maximal activation of the GEF and the lower value represents the loss of GEF function. The basal level of unstimulated GEF activity is set as corresponding to a gamma value of 0.1.

The effect of different concentrations of Cetuximab and cmp4 was simulated by perturbing the reference parameterization (4^th^ column of Supplementary Table S2) of the model as reported in Supplementary Table S1. In detail, the maximal action that could be obtained by an inhibitor acting by rescuing EGFR hyperactivation was simulated by dividing K_M,10_ and K_M,11_ by 10. The effectiveness of Cetuximab-like inhibitors was analyzed by a PSA performed by multiplying the V_max_ of GEF-mediated exchange reactions (R_10_ and R_11_) for a parameter gamma. This parameter was varied in the range 0-1, where the absence of EGFR stimulation is represented by a 0.1 value. cmp4 (at 100 µM) expected effect was simulated by multiplying K_M,10_ and K_M,11_ by 0.5, k_2-5_ by 0.5, and k_6_ by 0.23 (yielding a half amount of Ras-GTP-Eff complex formation).

## 3 Results

### 3.1 cmp4 Binds to both GDP-Bound Wild Type and G13D Mutated HRas Proteins

**FIGURE 1:**
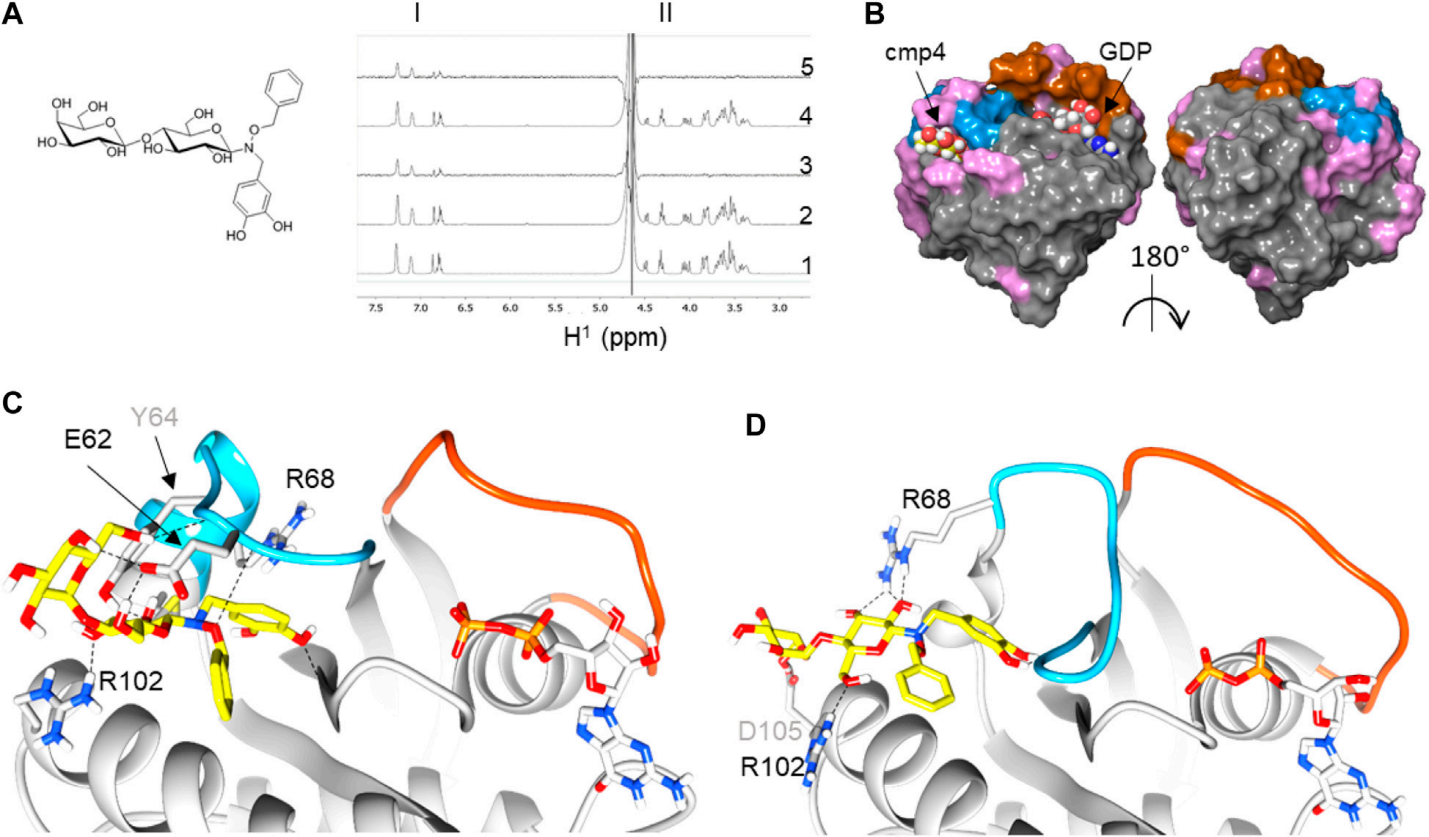
Reconstruction of a binding pose for cmp4 on wild-type and G13D mutated HRas-GDP complexes. **(A)** Chemical structure of cmp4; **(B)** NMR analysis traces report including aromatic resonance (region I), sugar resonance, and aliphatic CH_2_ (region II): 1)^1^H NMR spectrum of 1 mM cmp4; 2) ^1^H NMR spectrum of a sample containing 1 mM cmp4 and 50 μM HRas-GDP wt; 3) STD-NMR spectrum of a sample containing 1 mM cmp4 and 50 μM HRas-GDP wt; 4) ^1^H NMR spectrum of a sample containing 1 mM cmp4 and 50 μM HRas^G13D^-GDP; 5) STD-NMR spectrum of a sample containing 1 mM cmp4 and 50 μM HRas^G13D^-GDP. **(C)** Docking pose of cmp4 on PDB structure of HRas-GDP (PDB ID: 4q21). The image shows switch I (red), switch II (blue) and displays in pink the residues of Ras that undergo significant chemical shift perturbations after binding with cmp4 (Sacco et al., 2011); **(D,E)** Molecular detail of the selected pose of cmp4 on: **(D)** HRas-GDP (PDB ID: 4q21); **(E)** HRas^G13D^GDP (PDB ID:6dzh). Ras residues that are directly involved in binding with cmp4 are indicated. The backbone in the Switch I region is colored in red while the backbone in the Switch II region is colored in blue. The GDP nucleotide (grey) and cmp4 (yellow) are drawn in sticks. Heteroatoms are in red (oxygen) and blue (nitrogen).

By NMR analysis, we previously showed that cmp4 (Figure 1A) binds HRas-GDP (Sacco et al., 2011) and –mainly through its aromatic moiety–in a binding pocket located between the α2-(Switch II) and α3-helices (Figure 1B). Flexible docking indicates that cmp4 binds to an extended Switch II pocket (here referred to as SII-EP) of HRas and KRas (Figures 1C,D; Supplementarys S1A,B and Table S3). This pocket partially overlaps with the Switch II groove (SII-G) identified on KRas by structural analysis in Gentile et al. (2017) (Supplementary Figures S2A,D,G). Results of an STD-NMR analysis of HRas-GDP with cmp4 (Figure 1B) and additional data collected on similar compounds (Palmioli et al., 2009b; Palmioli et al., 2009a; Palmioli et al., 2017; Colombo et al., 2010; Sacco et al., 2011) support the pivotal role of the benzyl group and the pyrocatechol group for Ras binding. We used these results to filter the top 10 poses in this and other docking experiments.

cmp4 is a much bulkier molecule than the compound reported in Gentile et al. (2017) and occupies a larger pocket than the one there described (Supplementary Figures S2B,E,H), protruding towards the Gly^12^ P-loop. The cmp4 pyrocatechol group, as obtained in all of the docking best scoring poses, is much farther from this loop than the G12C binding compounds first described to target an allosteric switch II pocket (Ostrem et al., 2013; Patricelli et al., 2016) (Supplementary Figures S2C,F,I). Notably, catechol interacts with residues not only in α2-(switch II) (Glu^62^, Tyr^64^, Arg^68^) and α3-helices (Tyr^96^, Arg^102^) but also with the backbone of Gly^10^ in the P-loop (see the ligand interactions plot in Supplementary Figure S1A).

STD analysis on HRas^G13D^ mutant protein saturated with cmp4 shows that cmp4 also interacts with the mutant protein. Flexible docking indicates that cmp4 maintains a similar positioning within the binding pocket of HRas^G13D^-GDP, or in KRas^G13D^-GDP as well, despite the partial switch II unfolding observed in the oncoprotein (Figures 1E,D; Supplementary Figures S1C,D). The top docking scores were slightly lower than obtained on the wild type proteins (Supplementary Table S3).

Since the pathological effect of Ras hyperactivity is due to the active, GTP-bound form, and the phenol-derived compounds occupying the Switch II groove (SII-G), identified by structural analysis in Gentile *et al.* (Supplementary Figure S1A), were reported to target Ras active form as well (Gentile et al., 2017), we also assessed whether the SII-EP pocket in GTP-bound HRas and HRas^G13D^. is available to cmp4 interaction. Due to the different conformation of Switch II, this pocket seems to be less available in the GTP-bound complex (Supplementary Figure S3), leading to a maximal docking score decreased in comparison to that observed in the GDP-bound form (Supplementary Table S3), but still consistent with data previously reported for compounds binding to analogous pockets (Ostrem et al., 2013; Lito et al., 2016; Patricelli et al., 2016).

### 3.2 cmp4 Inhibits the Intrinsic and GEF Mediated-Nucleotide Dissociation and Exchange on Wild Type and G13D Mutated HRas in a Dose-Dependent Manner

**FIGURE 2:**
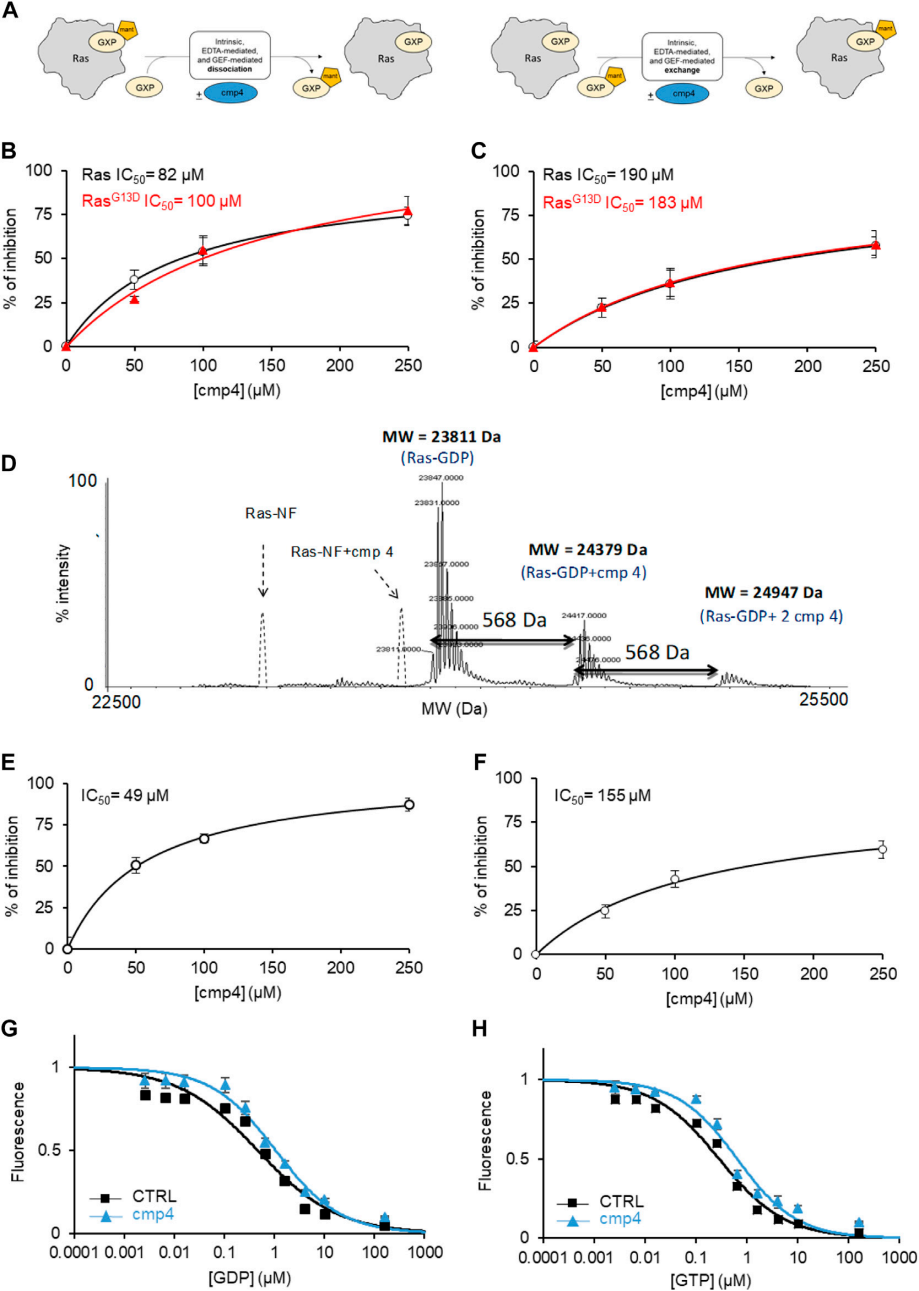
cmp4 counteracts nucleotide dissociation from Ras complex. **(A)** Scheme depicting the different experiments for nucleotide dissociation or exchange, using nucleotides (GDP or GTP, namely GXP) conjugated with the fluorescent moiety MANT; **(B,C)** inhibitory efficacy of cmp4 on GEF-mediated nucleotide dissociation **(B,C)** exchange on HRas (black) and HRas^G13D^ (red). **(D)** Mass spectrometry analysis of HRas-GDP in presence of cmp4. The dashed peaks correspond to the expected positions of the nucleotide free-Ras and Ras complexed with cmp4 without GDP. **(E,F)** Inhibitory efficacy of cmp4 on intrinsic nucleotide dissociation **(E)** and exchange **(F)** on HRas^G13D^; the initial dissociation or exchange rate of each reaction was determined computing the first derivative at time 0 of the fitted curves reported in Supplementary Figure S4. **(G,H)** EDTA-mediated competition between mant-GDP loaded on H-Ras and free unlabelled GDP **(G)** or GTP **(H)**.

mant-GDP is a nucleotide analog whose fluorescence increases upon Ras binding. The decrease in fluorescence following incubation of the Ras-mant-GDP complex with an excess of unlabeled GTP allows us to follow nucleotide exit (Figure 2A, left) The increase in fluorescence obtained after incubation of a Ras-GDP complex with an excess of mant-GDP directly monitors nucleotide entry (Figure 2A, right). In the normal Ras activation cycle, the entry of a new nucleotide immediately follows the nucleotide exit.

We previously demonstrated that cmp4 interferes with the function of the exchange factor RasGRF1 on HRas (Sacco et al., 2011). Here we show that cmp4 inhibits the GEF-catalyzed nucleotide dissociation and exchange reaction on wild type and G13D mutated HRas with similar efficiency (Figures 2B,C; Supplementary Table S4). Supplementary Figures S4A–D show the actual dissociation and exchange curves. We used the initial rates of each reaction (mean of at least three independent experiments) for calculating the IC50 reported in Figures 2B,C and Supplementary Table S4. The inhibitory effect of cmp4 on both dissociation and exchange reactions is independent of the GEF hSos1 vs. RasGRF1, (Sacco et al., 2011) and of the entering nucleotide, GDP or GTP (Supplementary Figure S5).

The docking results presented in Figure 1 suggest that cmp4 may form a stable Ras-nucleotide-cmp4 ternary complex, without promoting dissociation of the Ras-bound nucleotide, similar to peptide Ras inhibitors developed in our laboratory (Sacco et al., 2012b). The deconvoluted mass spectrum of 10 µM HRas-GDP in the presence of a 10-fold excess of cmp4 (Figure 2D) shows no signal corresponding to the nucleotide-free Ras/cmp4 complex. Except for a minor fraction of Ras-GDP binding a second inhibitor molecule at a low affinity, non-specific site, the HRas-GDP-cmp4 ternary complex is the most abundant species.

It was therefore of interest to monitor whether cmp4 can inhibit intrinsic (i.e., non GEF-catalyzed) nucleotide dissociation and exchange. We first tested the effect of cmp4 on HRas^G13D^, whose intrinsic nucleotide exchange rate is much higher than that of wild-type HRas (Palmioli et al., 2009b; Smith et al., 2013; Hunter et al., 2015; Johnson et al., 2019). Supplementary Figures S4E–H reports the actual dissociation and exchange curves. The inhibitor efficiently reduces the abnormally fast intrinsic nucleotide dissociation and exchange reactions on HRas^G13D^ in a dose-dependent manner (Figures 2E,F). The inhibitory effect is also appreciable on the intrinsic activities of wild type HRas, which are very slow *per se* (see the inserts in Supplementary Figures S4E,G).

Titration with unlabeled GDP and GTP of a HRas-mant-GDP complex in the presence of EDTA allows monitoring whether a drug alters the affinity for the entering nucleotide. Figures 2G,H indicate that cmp4 alters the entry of both nucleotides without discriminating between GDP and GTP, unlike the SII-P binding molecules described by Ostrem et al. (2013).

These results suggest that cmp4 binding to the Switch II extended pocket (SII-EP) counteracts nucleotide release, even in conditions favoring nucleotide release, as observed in HRas^G13D^ (Johnson et al., 2019), and/or in the presence of EDTA or a GEF catalytic domain.

### 3.3 cmp4 Reduces the Affinity of HRas-GDP for RasGRF1 and Raf1 Ras Binding Domain in a Dose-Dependent Manner

**FIGURE 3:**
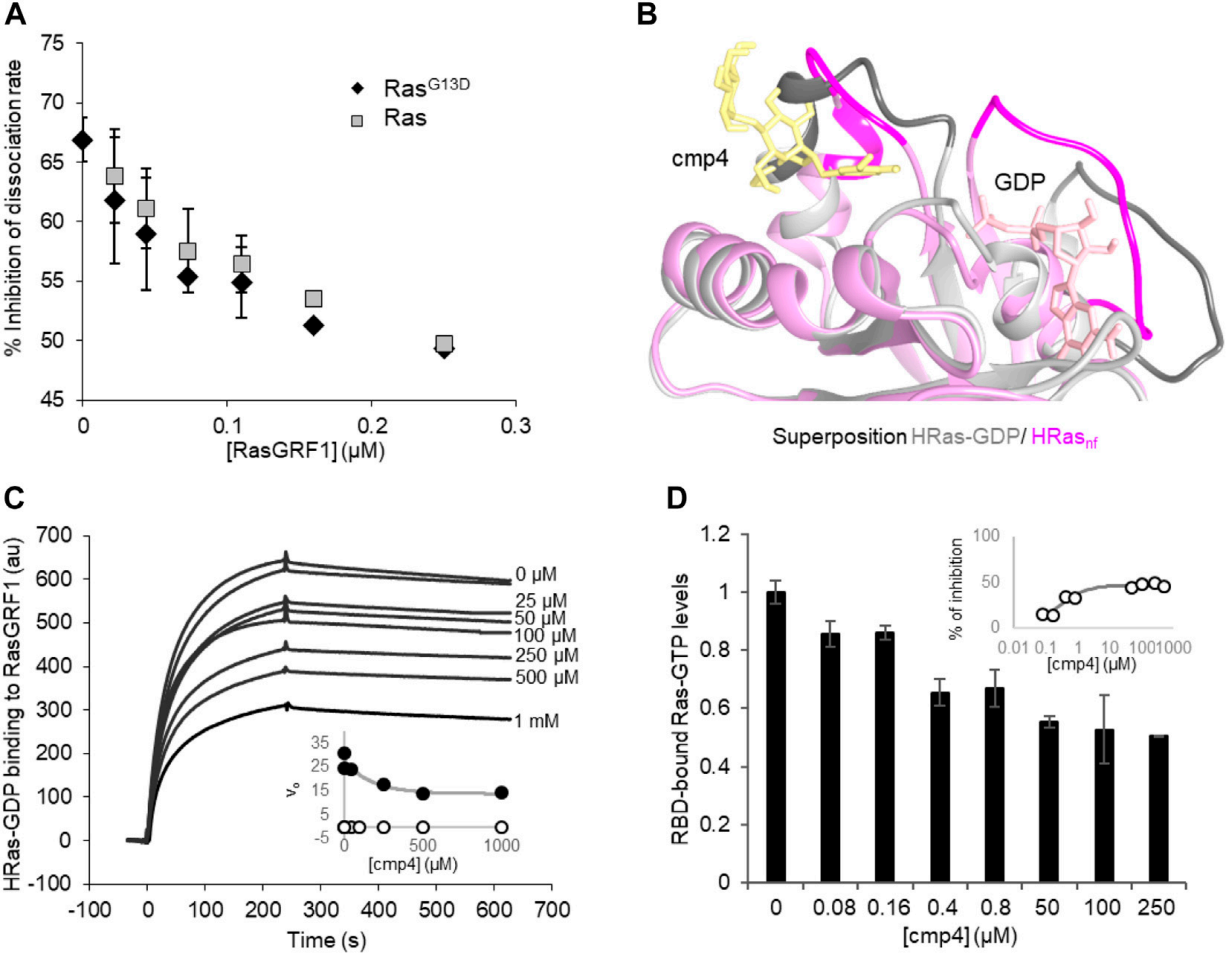
cmp4 affects HRas binding to GEF (RasGRF1) and effector (Raf1-RBD) in a dose-dependent manner. **(A)** Inhibition of nucleotide dissociation rate on both HRas and HRas^G13D^ (1 μM) in the presence of 100 μM cmp4 and increasing concentrations of RasGRF1 (range 0.01–0.25 μM). **(B)** Best fitting pose of cmp4 on HRas-GDP (pink) was superimposed to the structure of nucleotide-free Ras (HRas_nf_, in grey) from the crystal structure of the hSos1 catalytic domain associated with HRas (PDB ID: 1bkd). Switch I and II regions are stained darker. GDP is in pink, cmp4 in yellow; **(C)** Biacore-based direct measurement of 0.5 μM GEF (GST-RasGRF1) binding to His-HRas-GTP in the presence of increasing concentrations of cmp4 (25–1000 μM). In the insert kinetics analysis of RasGRF1 binding to HRas-GDP in the presence of different concentrations of cmp4, relative to SPR curves. All points for initial association rate (v_on_, closed symbols, v_off_, open symbols) were fitted respectively to a nonlinear ‘growth-sigmoidal Hill’ curve (*n* = 1), which is reported in the graph as a thin line; **(D)** Levels of HRas-GTP bound to a Ras binding domain (RBD) of Raf1 in the presence of increasing concentrations of cmp4 (range 0–500 μM), detected with the G-LISA^®^ kit (Cytoskeleton, Inc. BK131). Data were normalized to Ras-GTP levels measured in the absence of cmp4 (control). All data are significant at 99%, as calculated by Student’s t-test in comparison to control. In the inset, the percentage of inhibition of Ras-GTP bound to RBD as a function of cmp4 concentration, relative to the G-LISA experiment. All points were fitted respectively to a nonlinear ‘growth-sigmoidal Hill’ curve (*n* = 1).

The inhibitory efficiency of cmp4 on the nucleotide dissociation rate on both wild type and G13D mutated HRas decreases with increasing RasGRF1 concentration (Figure 3A), suggesting that the GEF could force the nucleotide dissociation even on cmp4-bound Ras, counteracting the inhibitor action. In order to bind the GEF catalytic domain with the highest affinity, HRas has to undergo a conformational change that allows nucleotide release (Boriack-Sjodin et al., 1998), as evidenced by the superposition of HRas structures respectively in GDP-bound and nucleotide-free Sos1_cat_-bound form (Figure 3B). Notably, the same kind of interaction is also envisioned for the catalytic domain of RasGRF1, due to homology with Sos1 (Freedman et al., 2006).

SPR binding experiments analyzed the interaction between Ras and GEF in the presence of increasing concentrations of cmp4 (Figure 3C). cmp4 affects GEF (RasGRF1) binding to Ras-GDP in a dose-dependent manner, with an estimated EC_50_ of 170 μM. In particular, cmp4 dose-dependently reduces the association rate, and so the k_on_ of the interaction (Figure 3C, inset), suggesting that the compound reduces the formation of the Ras/GEF complex, a key intermediate in Ras activation cycle. This finding agrees with the observation that cmp4 stabilizes the nucleotide-bound HRas conformation by bridging Switch I and Switch II (Figure 1D). This stabilized connection between Switch I and II would make Ras more refractory to the formation of the high-affinity complex with the GEF and to its catalytic action (Boriack-Sjodin et al., 1998).

The aberrant mitogenic signaling in Ras-driven cancer cells largely depends on the increased recruitment of the downstream effectors Raf1, from the constitutively active Ras oncoproteins (Metcalfe et al., 1993; Warne et al., 1993). Accordingly, molecules disrupting Ras/Raf1 association block KRas downstream signaling and impair Ras-mediated tumorigenic proliferation (Waldmann et al., 2004; Athuluri-Divakar et al., 2016; Trinh et al., 2016; Liu et al., 2017; McGee et al., 2018; Wiechmann et al., 2019). To assess the ability of cmp4 to affect Ras-GTP/Raf1 binding, we performed an ELISA assay with increasing concentrations of cmp4 (from 0 to 500 µM). Figure 3D shows that cmp4 reduces in a dose-response manner the amount of Ras-GTP complex bound to the effector Ras binding domain of Raf1 (RBD-Raf1) with an EC_50_ value of about 0.45 μM (IC_50_ about 250 μM, Figure 3D, insert).

### 3.4 cmp4 Reduces Cell Proliferation and MAPK Activation in KRas^G13D^ Expressing Cancer Cells

KRas–the predominantly Ras isoform mutated in cancer–presents a different amino acid in front of the binding pocket (glutamine instead of histidine in position 95) and a more disordered Switch II region even in the active conformation (Johnson et al., 2019) when compared to HRas. Docking poses and their scores (Supplementarys Figures S1B,D; Table S3) suggest that the pocket in KRas and KRas^G13D^ is equally available for cmp4 binding, consistently with the inhibitory effect exerted by cmp4 on KRas-transformed mouse fibroblasts (Sacco et al., 2011).

**FIGURE 4:**
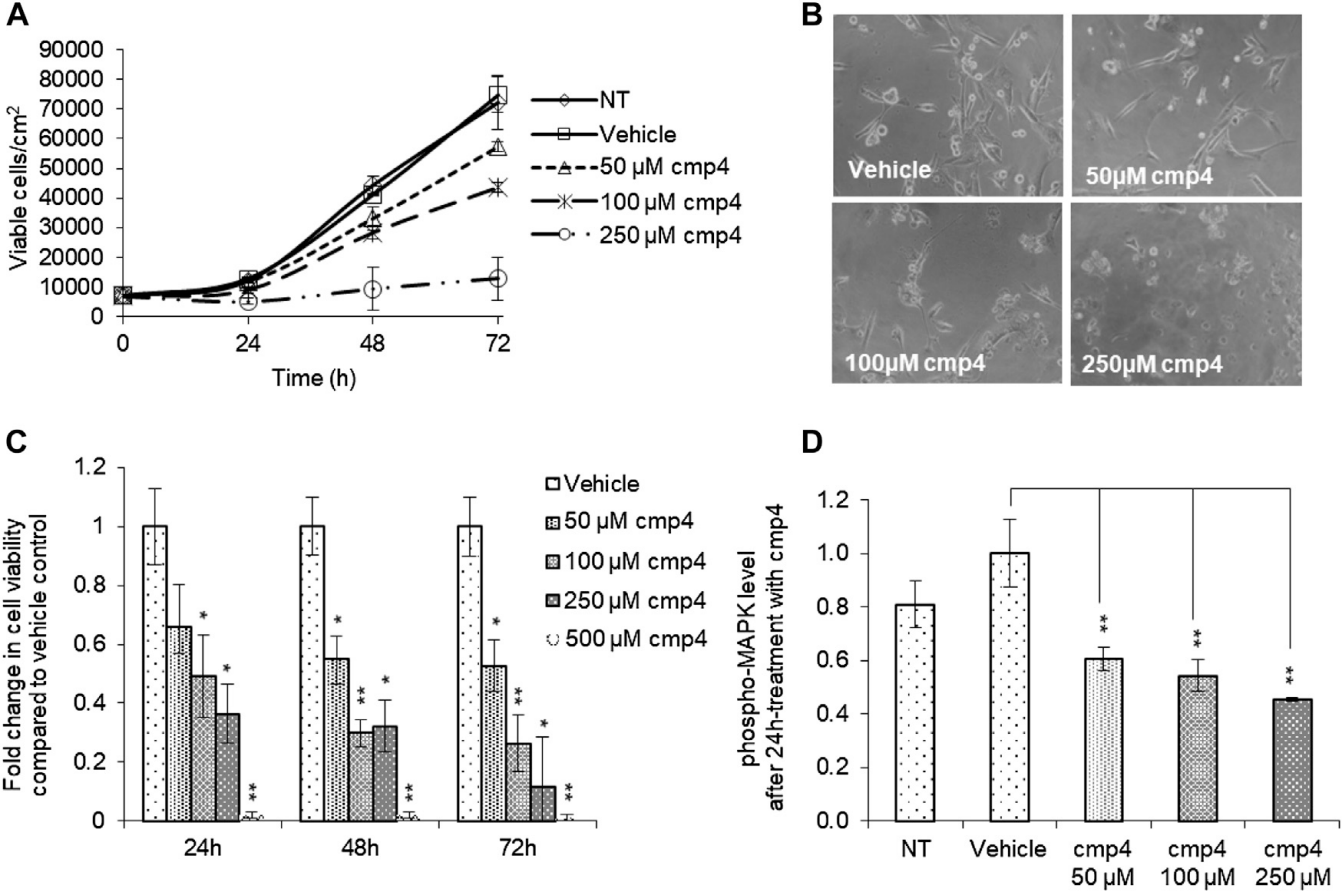
Effect of cmp4 on cell viability and Ras signaling of human breast cancer MDA-MB-231 cell line expressing KRas^G13D^. **(A)** Growth curves of MDA-MB-231 treated with increasing concentration of cmp4 or vehicle (deionized water) supplemented in the growth medium. After 24, 48, and 72 h of treatment cells were trypsinized and counted with a Burker chamber. **(B)** Microscopy analysis of MDA-MB-231 treated for 48 h with different concentrations of cmp4. **(C)** Cell viability of MDA-MB-231 cells treated with increasing concentration of cmp4, or vehicle (deionized water) for 24, 48, and 72 h as measured by MTT assay; data were normalized on cells treated with vehicle imposed as equal to 1. **(D)** Phosphorylated MAPK level in cell lysates from MDA-MB-231 cells no treated or 24 h-treated with cmp4 or vehicle. Data were normalized on the phospho-MAPK level in MDA-MB-231 treated with vehicle imposed as equal to 1. Data shown are mean and standard deviation of two independent experiments, each performed in triplicate. Single and double asterisk above histograms indicates a statistical significance of 95% and 99% respectively, calculated by Student’s t-test in comparison to cells treated with vehicle.

Here we evaluated the effect of cmp4 on MDA-MB-231, human breast cancer cells expressing KRas^G13D^. cmp4 reduces the proliferation of MDA-MB-231 cells in a dose-dependent manner (Figure 4A, IC_50_ of about 125 µM at 72 h), causing a significant cell detachment (see microscopy images in Figure 4B). MTT assays (Figure 4C) show that cmp4 significantly affects the viability of MDA-MB-231 cells already after 24 h-treatment. The reduced proliferative potential of cells treated with cmp4 correlates with a dose-dependent decrease of the level of activated/phosphorylated mitogen-activated protein kinases (MAPKs), as revealed by an ELISA assay performed on cell lysates collected after 24-h treatment with cmp4 (Figure 4D). Since high doses of cmp4 were administered to the cells, due to its low Ras affinity, we cannot exclude that the inhibition of proliferation is ascribable to off-target effects. However, the correlation between MAPK and cellular proliferation is consistent with a predominant specific effect on Ras activity.

### 3.5 Validation of the Mechanism of Action of cmp4 in Isogenic Cell Lines Expressing Different KRas Oncoproteins

Different Ras mutants produce a spectrum of distinct phenotypic effects and may display a significant difference in their ability to respond to therapies targeting the Ras pathway (Johnson et al., 2019). A recent computational model of the Ras activation cycle allows to explain and reproduce some of these different phenotypic traits, such as the peculiar sensitivity of *KRAS* mutants to Cetuximab, a drug targeting EGFR hyperactivation (McFall et al., 2019). Since results presented above and literature data (Sacco et al., 2011) indicate that cmp4 may interfere with multiple steps of the Ras activation cycle, we decided to use this model together with experiments on isogenic cell lines expressing different Ras mutant proteins to validate the mechanism of action of cmp4.

**FIGURE 5:**
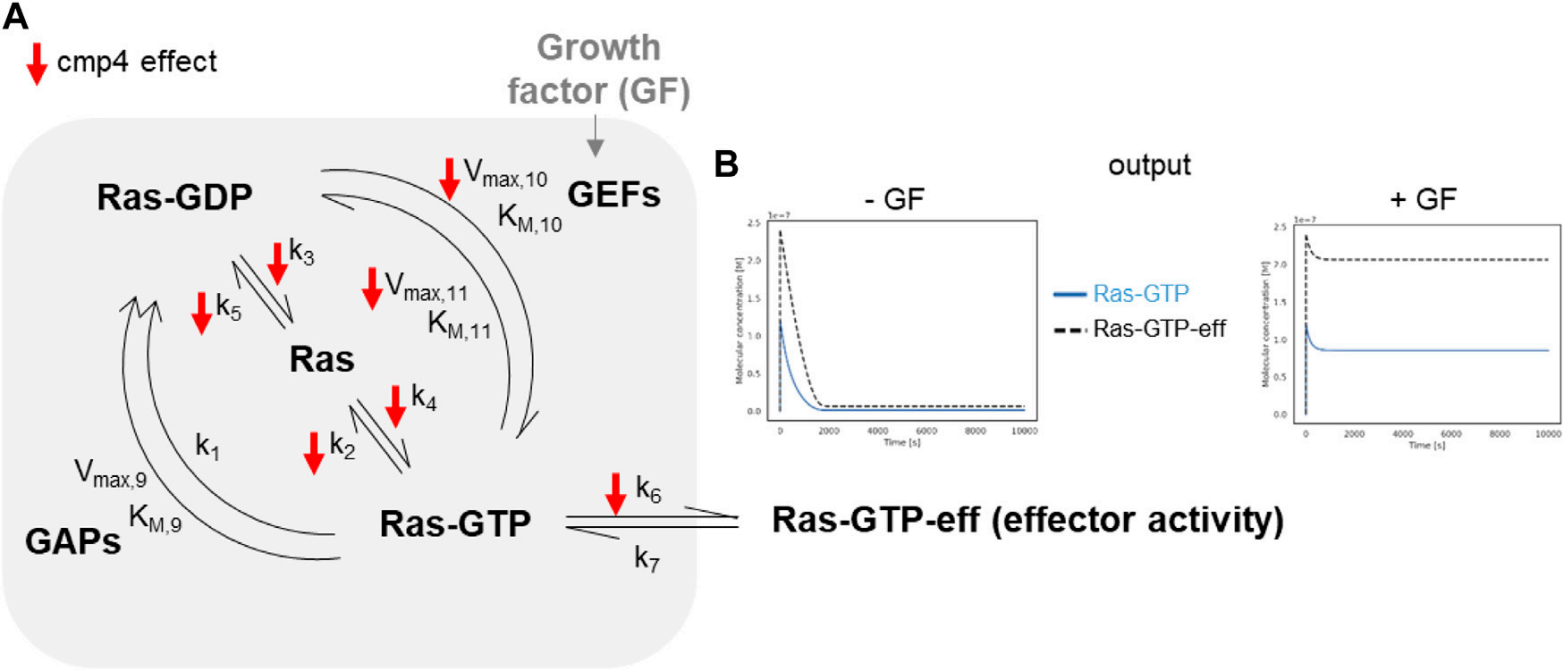
*In silico* modeling of Ras signalling network. **(A)** Scheme depicting the species and the reactions constituting the mathematical model. The parameters affected by cmp4 are indicated by red arrows. **(B)** Example of the output obtained simulating growth factor unstimulated **(left)** and stimulated **(right)** conditions of a wild type system. The level of effector activity, that is the level of the complex Ras-GTP-eff, is evaluated as the steady-state value reached during the dynamic simulation.

The model of the Ras activation cycle (Figure 5A) consists of 11 reactions (Supplementary Table S1). The first 8 reactions follow the mass-action kinetics, with a single kinetic parameter, while reactions R_9_, R_10_, R_11_ follow the Michaelis-Menten kinetics and require two different parameters. Parameter values can be changed to tailor the model to different cell systems. Supplementary Table S2 reports parameters used in this paper, that have been partially modified compared to McFall et al. (2019), by taking into account recent literature (Johnson et al., 2019; Rabara et al., 2019) and our own data. GEF activation induced by the interaction of a Growth Factor with its cognate receptor (reaction not included in the model) is simulated by an abrupt increase (up to 10-fold) of the V_max_ of the GEF-catalyzed reactions, i.e., V_max,10_ and V_max,11_ (grey arrow pointing to GEF in Figure 5A). Figure 5B (left panel) reports the results of a simulation of virtual cells in the absence of growth factor stimulation. Starting from nucleotide-free Ras, a rapid association of Ras with the available nucleotides is observed (guided by the fast reactions R_4_ and R_5_), then the level of Ras-GTP (grey line) and of the Ras-GTP-effector complex (dotted line) reach a steady state over the course of the simulation, characterized by a low level for both the species. When V_max,10_ and V_max,11_ are increased (simulating growth factor stimulation, Figure 5B right panel), both Ras-GTP and the Ras-GTP-Effector complex reach a steady-state level that is higher than the basal level observed in the absence of growth factor. In the following, we will use the steady-state level of the Ras-GTP-Effector complex to estimate the proliferation state of the simulated cell lines and to compare simulated and experimental data.

**FIGURE 6:**
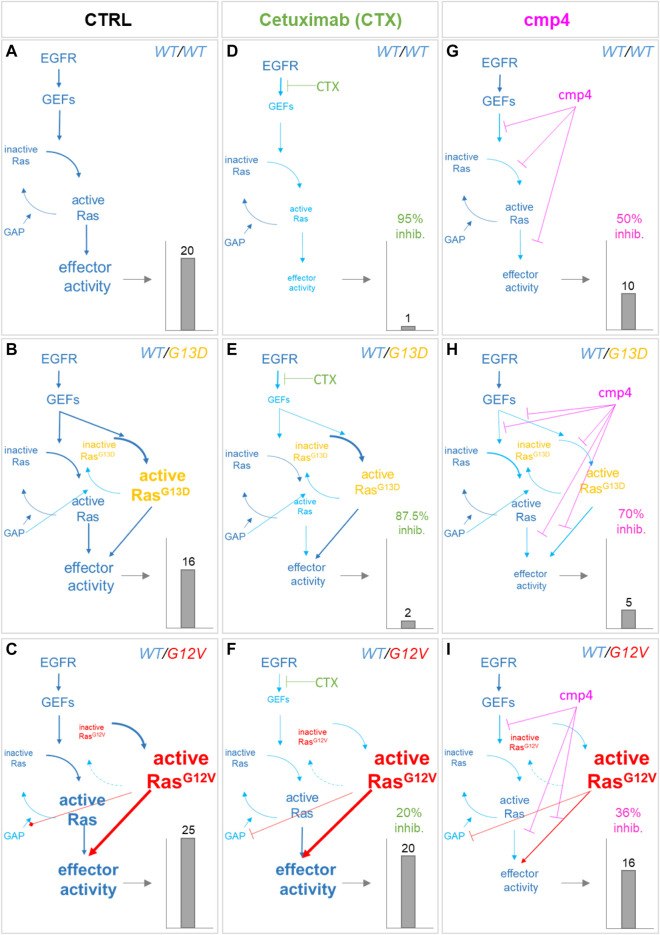
Simulation of the effect of Cetuximab and cmp4 on Ras signaling in human colorectal cancer SW48 isogenic cellular models. **(A–I)** *In silico* modeling of Ras signaling network in SW48 isogenic cellular models, expressing a hyper-activated EGF receptor mutant in combination with different Ras variants: either wild type Ras (**A**,**D**,**G**; SW48 *KRAS* ^*WT/WT*^) or KRas^G13D^ (**B**,**E**,**H**; SW48 *KRAS* ^*WT/G13D*^) or KRas^G12V^ (**C**,**F**,**I**; SW48 *KRAS* ^*WT/G12V*^). The different cellular systems were simulated under untreated condition (**A–C**; CTRL), or treated with the following drugs: Cetuximab **(D–F)**, used at an ideal concentration completely blocking EGFR activity, which represent the maximal effect obtainable with the single mechanism of action based on GEF-mediated nucleotide exchange inhibition; cmp4 **(G–I)**, used at the concentration of 100 µM, which is around IC_50_ for this compound on multilevel mechanisms of action (Supplementary Table S1). For each panel, the dimension and colour of the characters are indicative of the level of the components or their activity in the simulation. The resulting effector activity is illustrated as a histogram on the right of each panel, and its fold change normalized on wild type unstimulated cells is reported on top. For panels **(D–I)**, the inhibition efficacy is calculated with respect to the untreated corresponding model.

The small red arrows in Figure 5A indicate the steps within the Ras activation cycle affected by cmp4. They include the reactions describing the intrinsic association to, and dissociation from, the nucleotide (R_2_-R_5_), reactions describing association to the effector (R_6_), and GEF-mediated reactions allowing nucleotide exchange (R_10_ and R_11_). To study the effect of cmp4 on the Ras activation cycle we instantiated three different models representing a cell line endowed with a constitutively active EGFR mutant (EGFR^G719S^). This mutant receptor constitutively recruits GEFs to the plasma membrane causing an aberrant Ras activation. We simulated this mutation by imposing the maximal value for V_max,10_ and V_max,11_. The wild type cell line carries two wild type *KRAS* alleles, while two mutant cell lines express KRas^G13D^ and KRas^G12V^ in heterozygosis. Simulation of these virtual cell lines shows that the *KRAS* ^*WT/G12V*^ heterozygous mutant is the most aggressive based on the level of total KRas-effector complex, followed by the wild type and by the *KRAS* ^*WT/G13D*^ (Figures 6A–C). Although surprising at first sight, this result likely reflects the lower affinity of the KRas^G13D^ mutant protein for Raf1 (Johnson et al., 2019).

**FIGURE 7:**
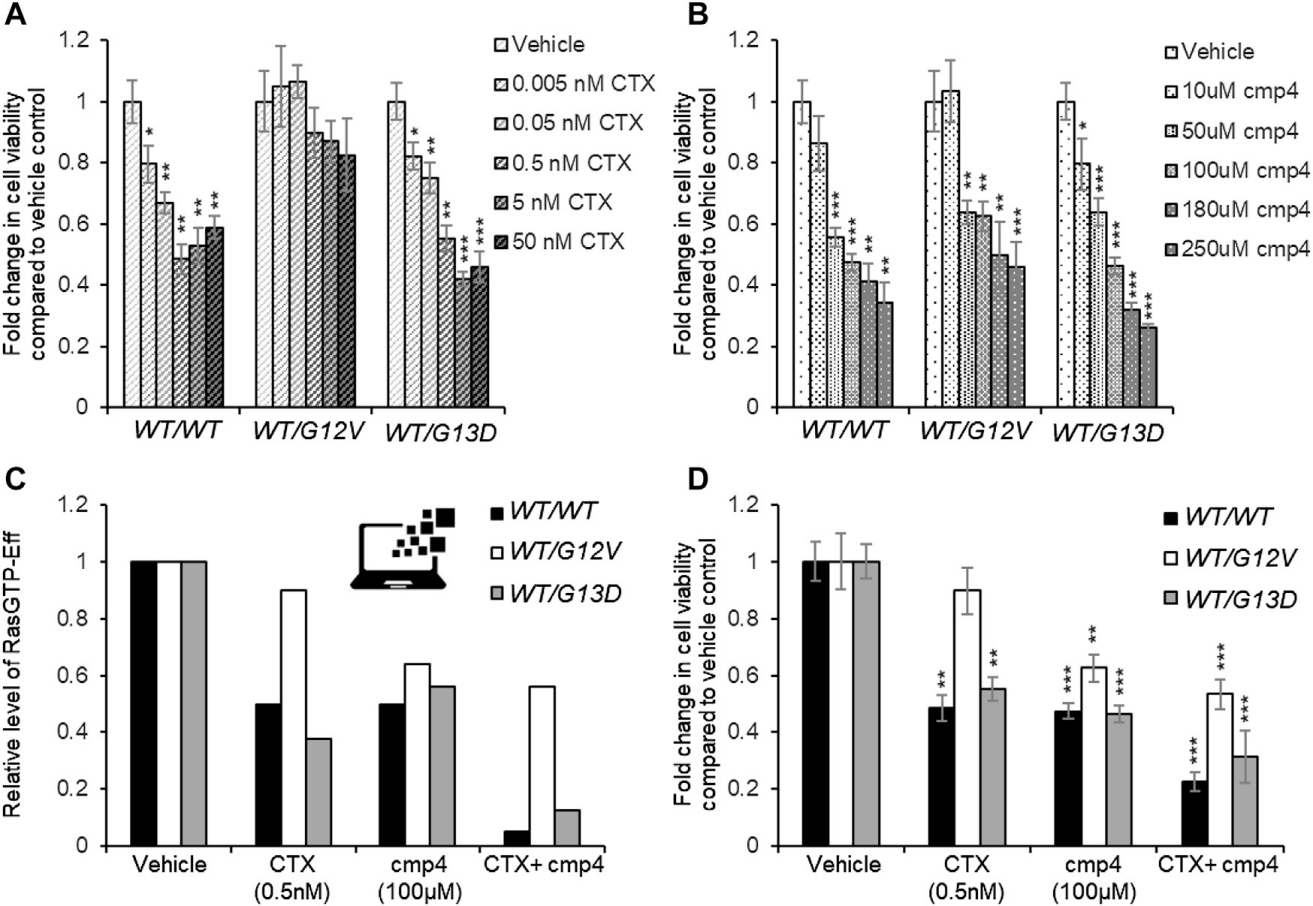
Effect of 72 h-treatment with cmp4 and/or Cetuximab (CTX) on cell viability of human colorectal cancer SW48 isogenic cell lines. **(A–C)** Relative cell viability of SW48 *KRAS* ^*WT/WT*^, SW48 *KRAS* ^*WT/G12V*^ *,* and SW48 *KRAS* ^*WT/G13D*^ cells treated for 72 h with different concentrations of CTX **(A)** or cmp4 **(B)**. **(C)** Results from the simulations of the SW48 *KRAS* ^*WT/WT*^, SW48 *KRAS* ^*WT/G12V*^ and SW48 *KRAS* ^*WT/G13D*^ mathematical models either untreated or treated with the following drugs: 0.5 nM CTX (corresponding to an inhibition of nearly 70% of GEF activity); 100 µM cmp4, which is around IC_50_ for this compound on multilevel mechanisms of action; a combination of both. **(D)** Relative cell viability of SW48 *KRAS* ^*WT/WT*^, SW48 *KRAS* ^*WT/G12V,*^ and SW48 *KRAS* ^*WT/G13D*^ cells treated for 72 h either with 0.5 nM CTX, 100 μM cmp4, or a combination of both. Data were normalized on cells treated with vehicle taken equal to 1. Single, double, and triple asterisk above histograms in **(A,B,D)** indicates a statistical significance of 95%, 99%, and 99.9% respectively, calculated by Student’s t-test in comparison to cells treated with vehicle.

As confirmed by our results (Figure 7A), the presence of the GAP-insensitive KRas^G12V^ mutant confers resistance to the treatment with Cetuximab (Burgess et al., 2003; Seshacharyulu et al., 2012). Computational results predict that the theoretical maximal effect exerted by Cetuximab (i.e., a complete reversion of GEFs activation) leads to a reduction of virtual proliferation (i.e., a reduction in the level of the Ras-GTP-effector complex) of 95% in the SW48 *KRAS* ^*WT/WT*^ model, of 87% in the SW48 *KRAS* ^*WT/G13D*^ model and only of 20% in the SW48 *KRAS* ^*WT/G12V*^ model (Figures 6D–F). These simulation results are consistent with Ras^G13D^ being responsive to GEFs action (Palmioli et al., 2009b; Smith et al., 2013; Johnson et al., 2019) and Supplementary Figure S4, whereas KRas^G12V^ is fully active even if GEFs are not activated (Supplementary Figure S6).

The potential inhibitory effect of cmp4 was tested on all the models, in the hypothesis that it could behave as a panRas inhibitor. The appropriate constants (Figure 5A) were modified with respect to the untreated case, by considering the biochemical effect induced by treatment with 100 µM cmp4 in the appropriate i*n vitro* assay (see Supplementary Table S1 for actual values used in simulation experiments)*.* Both experimental cell viability assays and simulation results indicate that all three virtual cell lines are sensitive to cmp4 (Figures 6G–I, 7B), the SW48 *KRAS* ^*WT/G12V*^ cell line being the less sensitive (Supplementary Table S5).

These results prompted us to test whether the combined use of both drugs could improve the pharmacological treatment of the G12V mutant. Simulation results indicate that the combined treatment is additive or nearly additive in the three cell line models SW48. The effect is striking in the *KRAS* ^*WT/WT*^ and *KRAS* ^*WT/G13D*^ models (Figure 7C, black and grey bars, respectively), but nevertheless noticeable also in the *KRAS* ^*WT/G12V*^ model, where complete inhibition of the EGFR cascade (i.e., leaving V_max,10_ and V_max,11_ at their basal level) has only a 10% effect on the level of the Ras-GTP-effector complex (Figure 7C, white bars). We fully confirmed these simulation results by measuring the inhibition in cell proliferation of the three cell lines treated with a combination of the two drugs (Supplementary Figure S7) and in particular with 0.5 nM Cetuximab (CTX), 100 μM, cmp4, or a combination of the two drugs (Figure 7D), validating the multi-level mechanism of action of cmp4 suggested by the molecular docking and biochemical assays described above.

## 4 Discussion

Reported success in the direct targeting of Ras proteins, long postulated as undruggable, has paved the way to the possible pharmacological inhibition of Ras in anti-cancer therapy. Best results, so far, were obtained with mutation-specific inhibitors, such as irreversible inhibitors binding mutant Ras^G12C^ proteins (Ostrem et al., 2013; Lito et al., 2016; Patricelli et al., 2016; Hansen et al., 2018b; Janes et al., 2018). These molecules target the SII-P allosteric cavity in the GDP-bound form and prevent the GEF-mediated nucleotide exchange and, indirectly, effector engagement. Notably, the *in vivo* efficacy of these inhibitors depends on the fact that Ras^G12C^ does not permanently remain in a GTP-bound form, likely because of relevant retained intrinsic GTPase activity (Hunter et al., 2015). Ras oncoproteins with impairment of both intrinsic and GAP-mediated GTP hydrolysis, such as Ras^G12R^, Ras^G12V^, and Ras^QL61^ (Hunter et al., 2015), would be refractory to this inhibitory action mechanism. Other promising compounds targeting a cryptic phenol-capturing groove near SII-P in both GDP- and GTP-bound forms of non-G12C Ras mutants were identified (Gentile et al., 2017). They are reversible inhibitors preventing the GEF-mediated nucleotide exchange and PI3K engagement, but they do not affect the binding to Raf1. These inhibitors seem particularly interesting for targeting the HRas isoform, which is a more potent activator of PI3K than KRas isoform (Yun et al., 1998). Although new powerful approaches for inhibiting Ras signaling in cancer have been recently developed (Gilardi et al., 2020; Shin et al., 2020) the challenge for the identification of inhibitors effective on the non-G12C pathological Ras variants is still open.

Here we show that cmp4 is a water-soluble pan-Ras inhibitor with a complex, multi-level mechanism of action. cmp4 is the product of rational design from a lead compound in which a 3,4-dihydroxyphenyl group (catechol) and a benzyloxy group, are interconnected by a linear linker (Palmioli et al., 2009b). cmp4 targets the SII-G pocket, as the compounds identified by Gentile et al. (2017). Since cmp4 is bulkier, it occupies a more extended region protruding towards the G12(P-)loop, here named SII-EP (Supplementary Figures S2E,H). The catechol group of cmp4 can undergo several different interactions with residues not only in α2-(switch II) (such as Glu^62^, Tyr^64^, and Arg^68^) and α3-helices (such as Tyr^96^ and Arg^102^), but also with the backbone of Gly^10^ in the P-loop (see ligand interactions plot in Supplementary Figure S1). Natural compounds containing a pyrocatechol group also target this pocket: 5-O-caffeoylquinic acid (5-CQA) takes contact with HRas-GDP through its aromatic caffeic acid moiety but is less efficient in inhibiting RasGRF1 binding (Palmioli et al., 2017). Although the residues in Switch II are the most affected upon cmp4 binding according to NMR analysis, the residues revealing a change in their chemical environment are more widespread along Ras protein (Sacco et al., 2011) suggesting that the binding of the compound could induce a deeper conformational rearrangement that cannot be reproduced by any docking protocol, in agreement with the effects observed for other compounds binding to this area (Ostrem et al., 2013; Gentile et al., 2017).

Treatment with cmp4 prevents intrinsic and GEF-mediated nucleotide exchange, both in wild type and in the G13D-mutated Ras protein, which is self-sufficient in nucleotide dissociation although remaining sensitive to GEF catalytic activity. In addition, cmp4 reduces Ras/Raf1 binding. This effect suggests that cmp4 is able to accommodate in the Switch II pocket of GTP-bound Ras proteins, either interfering with the hydrogen bonds network involved in stabilizing the State 2 Switch I conformation, required for Raf1 binding (Buhrman et al., 2007), or at least destabilizing the ordered Switch II conformation (R state) which allows high-affinity binding to Raf1. A more disordered T State is indeed adopted whenever α3 helix is shifted towards Switch II (Johnson et al., 2019). The presence of cmp4 in the SII-EP site could counteract the shift to the R state, which is characterized by a narrower pocket (Buhrman et al., 2010).

Simulation of the multi-level action mechanism of cmp4 in a computational model describing the Ras activation cycle in conditions designed to represent cells with a constitutively active EGF Receptor, suggests that the compound can work on different Ras oncoproteins, including KRas^G13D^ and KRas^G12V^. cmp4 effectively cooperates with compounds blocking the Ras signaling cascade at the level of the EGF Receptor, such as Cetuximab. A near additive effect is observed even in the presence of the Ras^G12V^ mutant that makes virtual and real cells insensitive to the inhibition of GEF activity resulting from treatments with Cetuximab. *In vitro* growth inhibition induced by cmp4 and Cetuximab (administered individually or in combination) on isogenic SW48-derived cell lines expressing different Ras mutant proteins fully confirm the simulation results.

## 5 Conclusion

With its multi-level mechanism of action that is only minimally superimposed with that of Cetuximab, cmp4 is a good candidate for medicinal chemistry efforts tailored at improving its currently unsatisfactory affinity for Ras proteins.

As a pan-Ras inhibitor, cmp4 is able to inhibit not only Ras oncoproteins but also the wild type variant when activated in a stimulus-dependent way. This would allow cmp4-based drugs to be effectively used in combination therapies with Cetuximab to reduce the proliferation of tumor cells expressing the constitutively activated RTK receptor, but also suggests certain cytotoxicity on proliferating cells in general, given that proliferation in mammalian cells is essentially promoted by Ras signaling. A low affinity is desired when dealing with treatments affecting wild-type Ras, in order to avoid general toxicity to non-proliferating cells, although the affinity of cmp4 still needs some improvement for this aim. It is noteworthy that the development of drugs specific for a pathogenic Ras variant could be achieved by adding chemical groups that can efficiently interact with the variant molecular features, such as the glutamate residue present in G12D or G13D KRas mutants, gaining in specificity and affinity for the targeted oncoprotein and allowing the administration of lower doses, ineffective on wild-type Ras proteins.

## Data Availability

The raw data supporting the conclusions of this article will be made available by the authors, without undue reservation.
