# Supplementary material for: The Multi-Level Mechanism of Action of a Pan-Ras Inhibitor Explains its Antiproliferative Activity on Cetuximab-Resistant Cancer Cells
Source: Front Mol Biosci. 2021 Feb 17;8:625979. doi: 10.3389/fmolb.2021.625979 (PMC7925909; doi:10.3389/fmolb.2021.625979)
Supplement: Supplementary file 1 [file datasheet1.pdf]

Supplementary Materials:

Supplementary Figures

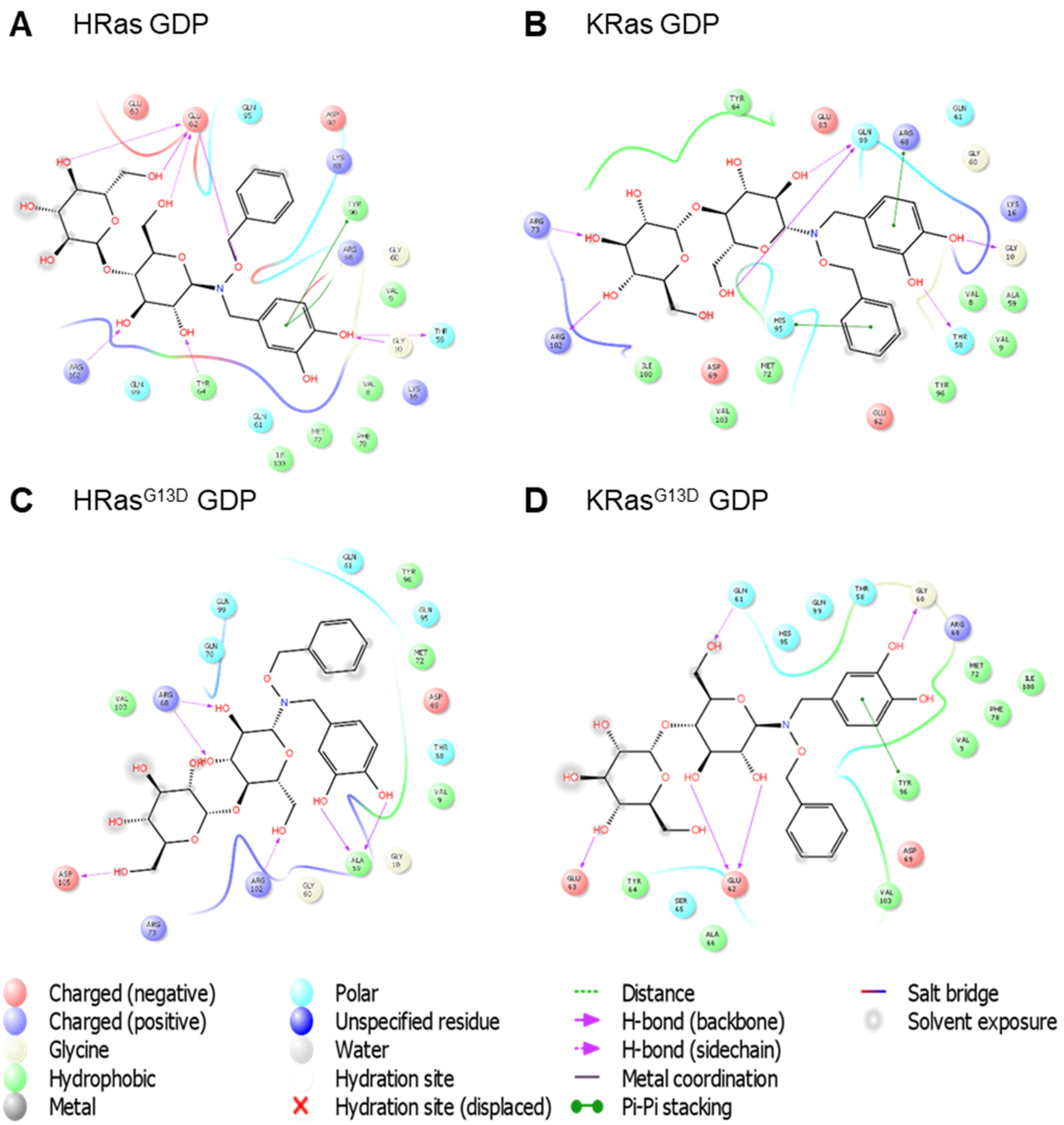

Supplementary Figure 1. cmp4 can bind wild-type and G13D mutated HRas and KRas isoforms in a similar manner. Ligand interaction plots of the poses obtained by flexible docking for cmp4 on the indicated structures, chosen according to available experimental hints. The structures used are as follows: (A) HRas-GDP (PDB ID:4q21); (B) KRas-GDP (PDB ID:4lpk); (C) HRas^{G13D}-GDP (PDB ID:6dzh); (D) KRas^{G13D}-GDP (PDB ID:4tqa).

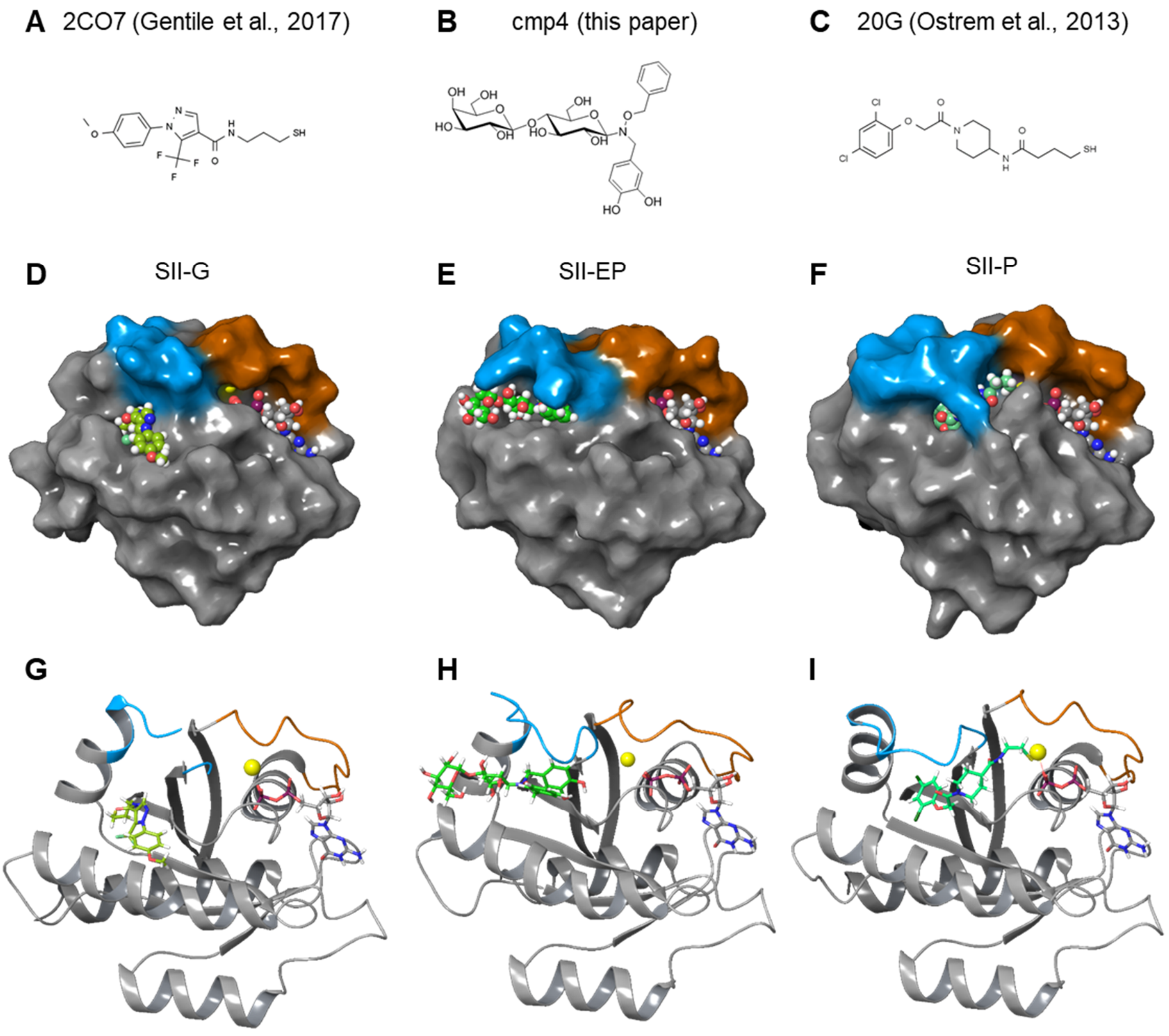

Supplementary Figure 2. Comparison of cmp4 pose on KRas-GDP (PDB ID:4lpk) with known inhibitors that accomodate in a Switch II pocket. (**A**) Structure of 2CO7 inhibitor by Gentile et al., 2017; (**B**) structure of cmp4; (**C**) structure of 20G compound by Ostrem et al., 2013. (**D**,**G**) Structure of KRas^{M72C}-GDP covalently bound to 2CO7 inhibitor on M72C residue (PDB ID:5vbm, Gentile et al., 2017): the inhibitor is bound in a so called SII-G pocket, which is described as a phenol accomodating groove; (**E**,**H**) cmp4 best fitting pose on KRas-GDP (PDB ID:4lpk) indicates that cmp4 is bulkier and occupies a larger pocket than 2CO7 molecule, identifying a pocket here named SII-EP; (**F**,**I**) structure of KRas^{G12C}-GDP covalently bound to a disulfide inhibitor (structure of the compound is shown in C) on G12C residue (PDB ID:4luc, Ostrem et al., 2013): the inhibitor is bound in a so called SII-P pocket, which is described as a Ras-GDP specific pocket. Proteins are shown as surface (**D**, **E**, **F**) or as cartoons (**G**, **H**, **I**). Switch I is coloured in red, Switch II in blue. GDP (grey carbon atoms) and inhibitors (green carbon atoms) are drawn in lines (**A**, **B**, **C**), in spheres (**D**, **E**, **F**) or in sticks (**G**, **H**, **I**).

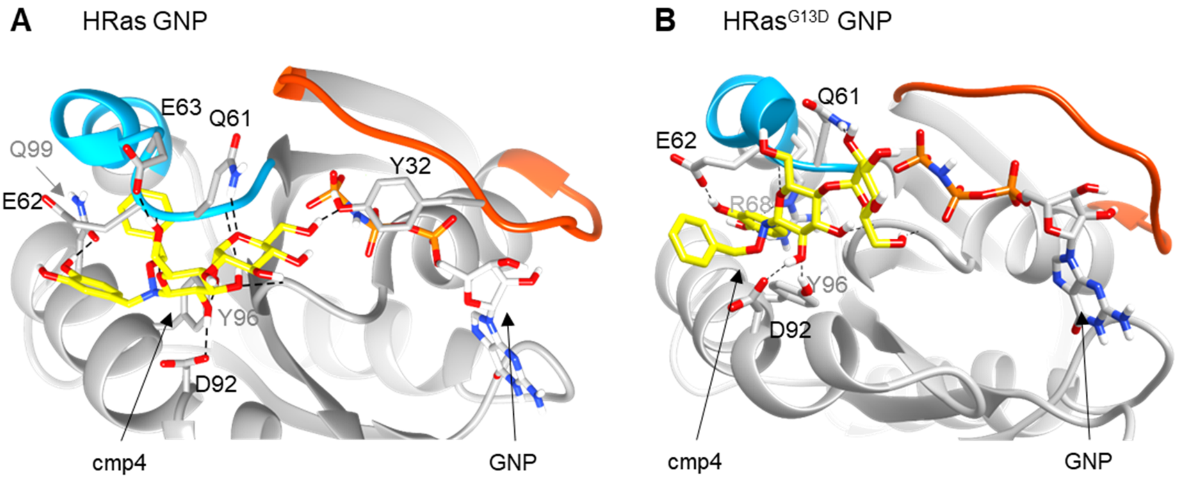

Supplementary Figure 3. The SII Extended Pocket is available in HRas and HRas^{G13D} in the active state. Best fitting docking poses for cmp4 (carbon atoms in yellow) on: (**A**) HRas-GNP structure (PDB ID: 5q21); (**B**) HRas^{G13D}-GNP (PDB ID:6e6p). Switch I is in red, Switch II in blue. cmp4 (carbon atoms in yellow) and GDP (carbon atoms in gray) are in sticks. Inhibitor interacting residues are labelled.

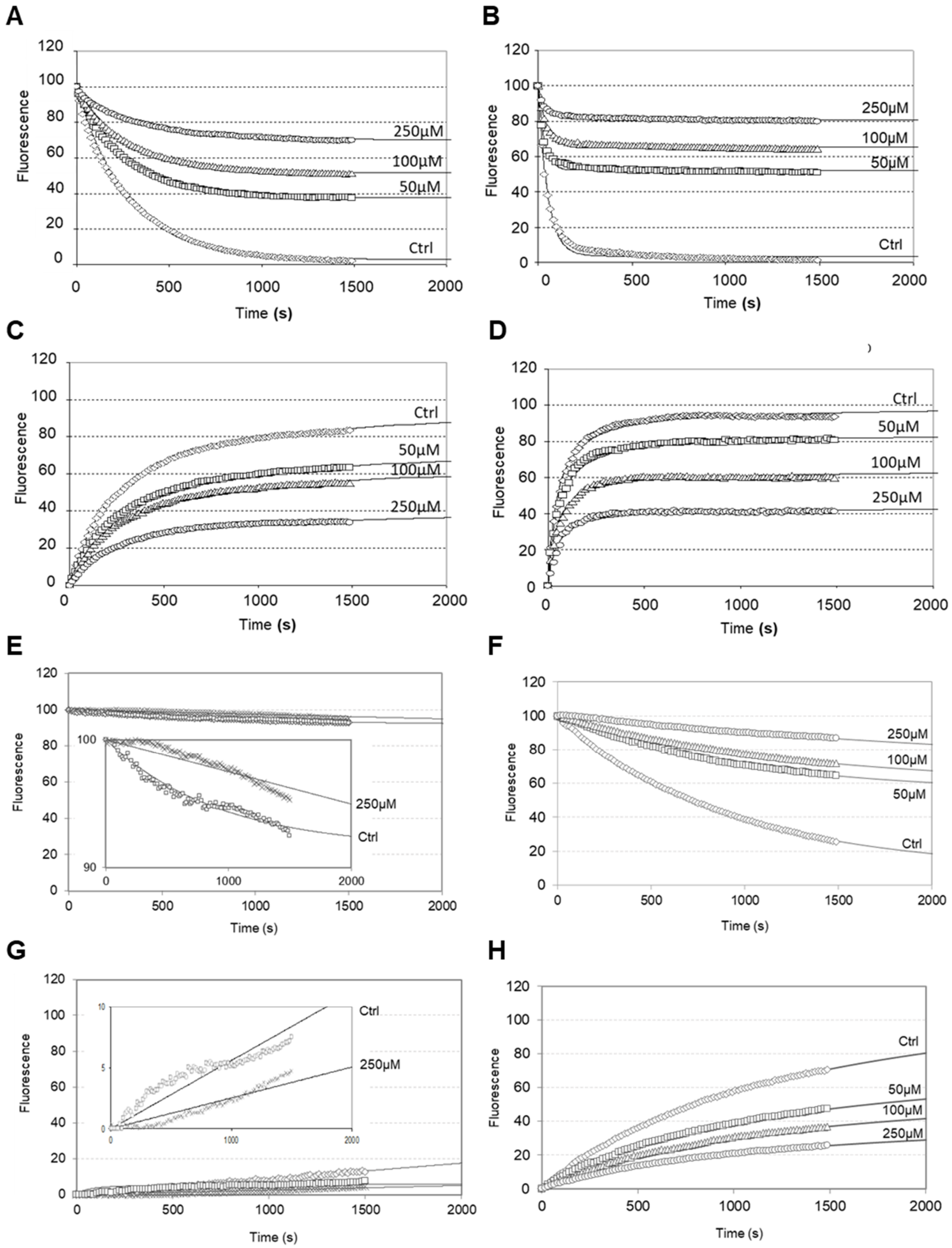

Supplementary Figure 4. cmp4 inhibits both intrinsic and GEF-mediated nucleotide dissociation and exchange on wild type and G13D mutated HRas. (**A**-**D**) Effect of cmp4 on GEF-catalyzed nucleotide dissociation and exchange of HRas (**A**,**C**) and HRas^{G13D} (**B**,**D**). The dissociation reactions were obtained by adding 200 µM GDP, 0.25 µM RasGRF1 and different concentrations of cmp4 to 1 µM HRas(wt or mutant)-mantGDP. The exchange reactions were obtained by adding 1.25 µM mantGDP, 0.0625 µM RasGRF1 and different concentrations of cmp4 to 0.25 µM

HRas(wt or mutant)-GDP. (**E**-**H**) Effect of cmp4 on intrinsic nucleotide dissociation and exchange of HRas (**E**,**G**) and $HRas^{G13D}$ (**F**,**H**). The dissociation reactions were obtained by adding 200 µM GDP and different concentrations of cmp4 to 1 µM HRas(wt or mutant)-mantGDP. The exchange reactions were obtained by adding 1.25 µM mantGDP and different concentrations of cmp4 to 0.25 µM HRas(wt or mutant)GDP. Exchange and dissociation curves were fitted respectively to a non linear 'growth-sigmoidal Hill' curve (n=1) and non linear 'exponential' curve, and were reported in graph as a thin line. In the graph the maximum value of relative fluorescence (100 on Y axis) represents the fully mantGDP-charged Ras status.

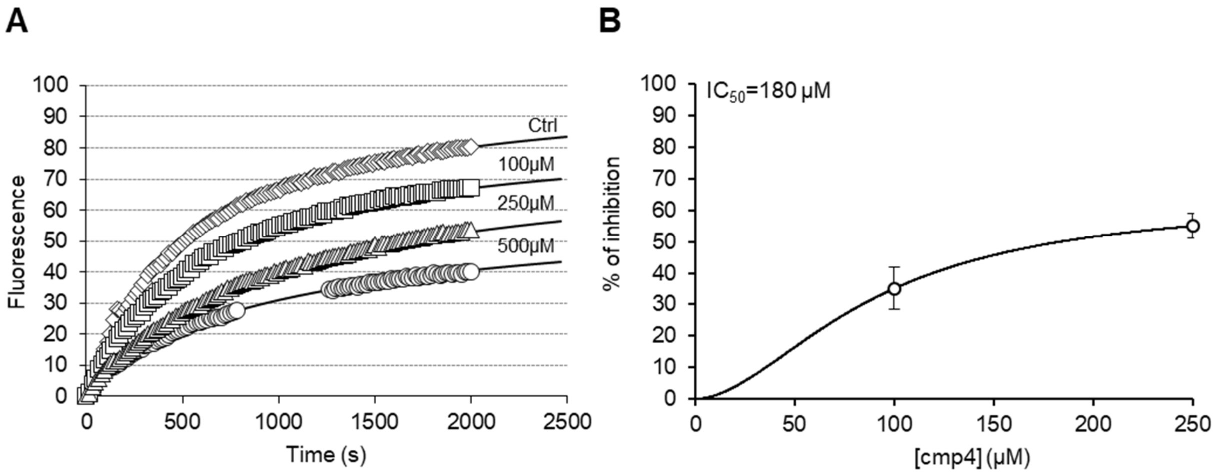

Supplementary Figure 5. Effect of cmp4 on GEF-catalyzed nucleotide exchange of HRas-GDP versus mantGTP fluorescent nucleotide. (**A**) The exchange reactions were obtained by adding 1.25 µM mantGTP, 0.0625 µM RasGRF1 and the indicated concentrations of cmp4 to 0.25 µM H-RasGDP. Fluorescence measurements were made with a Perkin-ElmerLS-45 luminescence spectrometer, monitoring the fluorescence each second for 2000 sec. Exchange curves were fitted to a non linear 'growth-sigmoidal Hill' curve (n=1), and were reported in graph as a thin line. In the graph the maximum value of relative fluorescence (100 on Y axis) represents the fully mantGTP-charged Ras status. (**B**) IC_{50} was calculated by plotting the dose response plot for the inhibition observed on the initial exchange rate of each reaction, as determined computing the first derivative at time 0 of the fitted curve.

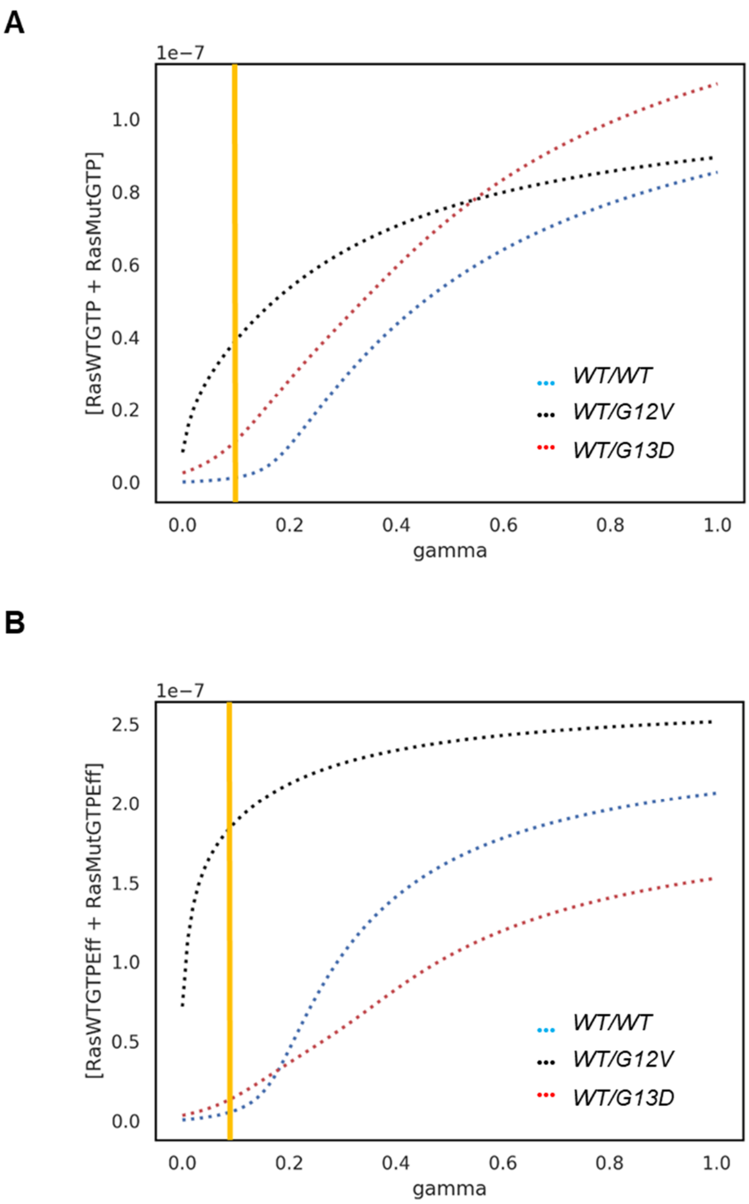

Supplementary Figure 6. Effect of GEF activity inhibition was analyzed by in silico simulation. The plots show the dynamics of free Ras(WT+MUT)GTP (**A**) and Ras(WT+MUT)GTP-Effector (**B**) complex level following a parameter sweep analysis (PSA) performed by varying the V_{max} of GEF-mediated exchange reactions for both wt Ras and Ras^{G13D} variant in the indicated mathematical model (i.e. R_{10} and R_{11}) by the scaling factor gamma indicated on the X-axis.

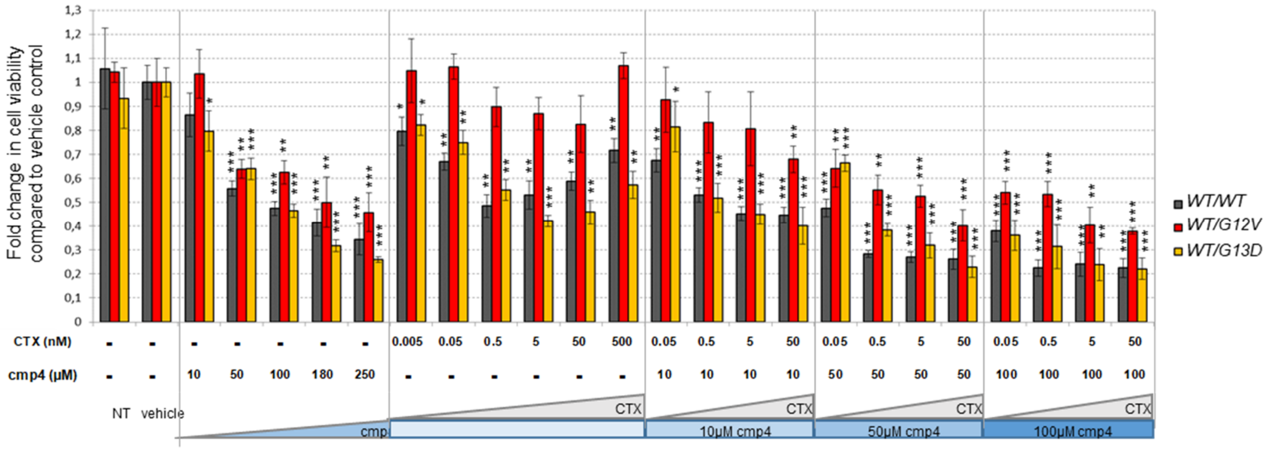

Supplementary Figure 7. Effect of 72h-treatment with cmp4 and/or Cetuximab (CTX) on cell viability of human colorectal cancer SW48 isogenic cell lines. (**A**-**C**) Relative cell viability of SW48 *KRAS*$^{WT/WT}$, SW48 *KRAS*$^{WT/G12V}$ and SW48 *KRAS*$^{WT/G13D}$ cells treated for 72 hours with different concentrations of CTX (**A**) or cmp4 (**B**) alone or in combination (**C**) measured with RealTime-Glo™ MT Cell Viability Assay (Promega). Data were normalized on cells treated with vehicle taken equal to 1. Single, double and triple asterisk above histograms indicates a statistical significance of 95%, 99% and 99.9% respectively, calculated by Student's t-test in comparison to cells treated with vehicle.

Supplementary Tables

Supplementary Table 1. Reactions included in the model. Alpha factors as shown were introduced to simulate the addition of single inhibitors. Combination of inhibitors were simulated by multiplying the indicated alpha factors, since their mechanisms of action are independent.

Reaction	Reagents	Products	100 µM cmp4	0.5 nM CTX
R_1	RasGTP	RasGDP	1	1
R_2	RasGTP	Ras	0.5	1
R_3	RasGDP	Ras	0.5	1
R_4	Ras + GTP	RasGTP	0.5	1
R_5	Ras + GDP	RasGDP	0.5	1
R_6	RasGTP + Eff	RasGTPEff	0.23	1
R_7	RasGTPEff	RasGTP + Eff	1	1
R_8	RasGTPEff	RasGDP + Eff	1	1
R_9	RasGTP	RasGDP $V_{max,9}$	1	1
		$K_{M,9}$	1	1
R_{10}	RasGDP	RasGTP $V_{max,10}$	0.5	0.2965
		$K_{M,10}$	1	1
R_{11}	RasGTP	RasGDP $V_{max,11}$	0.5	0.2965
		$K_{M,11}$	1	1

Supplementary Table 2. Species, reactions and parameters used in the mathematical model. Note that $K_{M,9}$, $K_{M,10}$ and $K_{M,11}$ were divided by the membrane partition simulating factor, that means by 250. (*) $V_{max,10}$ and $V_{max,11}$ were multiplied by 10 in SW48 models both for wild type and mutant Ras.

Reaction	Reagents	Products	Kinetic parameter (wild-type)	Ras^{G13D}	Ras^{G12V}
Intrinsic hydrolysis					
R_1	RasGTP	RasGDP	$k_1 = 3.5 \times 10^{-4}\ s^{-1}$	0.40	0.15
Intrinsic nucleotide dissociation					
R_2	RasGTP	Ras	$k_2 = 2.5 \times 10^{-4}\ s^{-1}$	3.63	0.80
R_3	RasGDP	Ras	$k_3 = 1.1 \times 10^{-4}\ s^{-1}$	3.63	0.31
Intrinsic nucleotide association					
R_4	Ras + GTP	RasGTP	$k_4 = 2.2 \times 10^{6}\ s^{-1}$	1	4.14
R_5	Ras + GDP	RasGDP	$k_5 = 2.3 \times 10^{6}\ s^{-1}$	1	2.27
Effector association					
R_6	RasGTP + Eff	RasGTPEff	$k_6 = 4.5 \times 10^{7}\ s^{-1}$	1	1
R_7	RasGTPEff	RasGTP + Eff	$k_7 = 3.6\ s^{-1}$	1	0.44
Intrinsic hydrolysis within Effector complex					
R_8	RasGTPEff	RasGDP + Eff	$k_8 = 3.5 \times 10^{-4}\ s^{-1}$	0.40	0.15
GAP-mediated hydrolysis					
R_9	RasGTP	RasGDP	$V_{max,9} = 3.24 \times 10^{-10}$ M/s	0.6	0
			$K_{m,9} = 2.3 \times 10^{-7}$ M	5	1
GEF-mediated nucleotide exchange					
R_{10}	RasGDP	RasGTP	$V_{max,10} = 7.8 \times 10^{-10}$ M/s (*)	1 (*)	1 (*)
			$K_{m,10} = 3.86 \times 10^{-4}$ M	1	1
R_{11}	RasGTP	RasGDP	$V_{max,11} = 1.4404 \times 10^{-10}$ M/s (*)	1 (*)	1 (*)
			$K_{m,11} = 3.0 \times 10^{-4}$ M	1	1

Supplementary Table 3. Best docking scores obtained for cmp4 on the indicated complexes. The best scores for each structure are reported.

Structure	PDB ID	Glide Gscore	Glide Emodel
**HRas**			
HRasGDP	4q21	-10.539	-144.004
HRasGNP	5p21	-9.262	-103.377
HRasGNP-Raf1RBD	4g0n	-8.956	-113.946
$HRas^{G13D}GDP$	6dzh	-8.569	-116.654
$HRas^{G13D}GNP$	6e6f	-8.023	-110.257
**KRas**			
KRasGDP	4lpk	-11.761	-146.808
$KRas^{G13D}GDP$	4tqa	-9.285	-111.533

Supplementary Table 4. Multilevel inhibitory effect of cmp4. IC_{50} values were calculated from different experimental readouts. FA, fluorimetric assay; SPR, surface plasmon resonance; GK, growth kinetics. nd, not determined.

Read-out	IC_{50} (µM)		Notes
	**wt**	**G13D**	
**Protein assays**			
intrinsic nucleotide dissociation	nd	50	FA
Intrinsic nucleotide exchange		150	FA
GEF-mediated nucleotide dissociation	80	100	FA
GEF-mediated nucleotide exchange	190	180	FA
Ras-GEF interaction	115	nd	SPR
Ras-RBD interaction	100	nd	G-LISA^{TM}
**Cellular models**			
cell proliferation (MDA-MB231)	nd	70	GK
cell viability (SW48)	80	85	RealTime-Glo^{TM} MT
		(IC_{50} G12V: 180)	

Supplementary Table 5. IC_{50} and EC_{50} values were calculated for 72h-treatment with cmp4 and/or Cetuximab on cell viability of human colorectal cancer SW48 wt and isogenic cell lines knock in for the oncogenic $KRas^{G12V}$ and $KRas^{G13D}$.

Structure	IC_{50}	EC_{50}
**cmp4 (µM)**		
SW48	80	30
SW48G12V	180	40
SW48G13D	85	40
**Cetuximab (nM)**		
SW48	0.25	0.01
SW48G12V	no effect	no effect
SW48G13D	0.4	0.05
